# Capsaicin binds the N-terminus of Hsp90, induces lysosomal degradation of Hsp70, and enhances the anti-tumor effects of 17-AAG (Tanespimycin)

**DOI:** 10.1038/s41598-023-40933-9

**Published:** 2023-08-23

**Authors:** Chaitanya A. Patwardhan, Vamsi Krishna Kommalapati, Taoufik Llbiyi, Digvijay Singh, Eyad Alfa, Anatolij Horuzsko, Hasan Korkaya, Siva Panda, Christopher A. Reilly, Vladimir Popik, Ahmed Chadli

**Affiliations:** 1 Georgia Cancer Center at Augusta University (Formerly Medical College of Georgia), 1410 Laney Walker Blvd, CN-3313, Augusta, GA 30912 USA; 2 Department of Chemistry and Biochemistry, Augusta University, Augusta, GA 30912 USA; 3 Department of Pharmacology and Toxicology, Center for Human Toxicology, College of Pharmacy, University of Utah, Salt Lake City, UT 84112 USA; 4 Department of Chemistry, University of Georgia, Athens, GA 30602 USA

**Keywords:** Protein folding, Structural biology, Cancer therapy

## Abstract

Heat shock protein 90 (Hsp90) and its co-chaperones promote cancer, and targeting Hsp90 holds promise for cancer treatment. Most of the efforts to harness this potential have focused on targeting the Hsp90 N-terminus ATP binding site. Although newer-generation inhibitors have shown improved efficacy in aggressive cancers, induction of the cellular heat shock response (HSR) by these inhibitors is thought to limit their clinical efficacy. Therefore, Hsp90 inhibitors with novel mechanisms of action and that do not trigger the HSR would be advantageous. Here, we investigated the mechanism by which capsaicin inhibits Hsp90. Through mutagenesis, chemical modifications, and proteomic studies, we show that capsaicin binds to the N-terminus of Hsp90 and inhibits its ATPase activity. Consequently, capsaicin and its analogs inhibit Hsp90 ATPase-dependent progesterone receptor reconstitution in vitro*.* Capsaicin did not induce the HSR, instead, it promoted the degradation of Hsp70 through the lysosome-autophagy pathway. Remarkably, capsaicin did not induce degradation of the constitutively expressed cognate Hsc70, indicating selectivity for Hsp70. Combined treatments of capsaicin and the Hsp90 inhibitor 17-AAG improved the anti-tumor efficacy of 17-AAG in cell culture and tridimensional tumor spheroid growth assays using breast and prostate cancer models. Consistent with this, in silico docking studies revealed that capsaicin binding to the ATP binding site of Hsp90 was distinct from classical N-terminus Hsp90 inhibitors, indicating a novel mechanism of action. Collectively, these findings support the use of capsaicin as a chemical scaffold to develop novel Hsp90 N-terminus inhibitors as well as its ability to be a potential cancer co-therapeutic.

## Introduction

The chaperome is composed of chaperone and co-chaperone proteins that safeguard cellular proteostasis and thus plays a key role in determining cancer phenotypes. Cancer cells over-express molecular chaperones and co-chaperones, including Hsp90, Hsp70, Hsp90/Hsp70 organizing protein (HOP), Hsp40, p23, and CDC37, that are necessary to fold mutant proteins and other highly expressed proteins^1–3^. Early studies have shown that heat shock protein 90 (Hsp90) is enriched in dynamic complexes with co-chaperones in tumor tissues compared to normal tissue^4,5^. Consistent with this, recent findings revealed the existence of a more complex molecular rewiring of the Hsp70 and Hsp90 chaperone networks to form a highly integrated complex, referred to as the epichaperome, in cancer cells. This pathologic protein–protein interaction network has been shown to promote cancer cell survival and aggressiveness of certain tumors^6^. The epichaperome may also be amenable to drug targeting^7^.

Hsp90 has been recognized as a therapeutic target for cancer therapy for decades^1^. Extensive efforts have led to the discovery of multiple Hsp90 inhibitors. Compounds of a diverse chemical nature, including geldanamycin derivatives, resorcinol group-containing compounds, purine and purine-like analogs, and others, have been established as potent inactivators of Hsp90 through binding to the ATP binding pocket in the N-terminal domain. However, many of these agents induce the cellular heat shock response (HSR), which is characterized by the up-regulation of the anti-apoptotic proteins, Hsp70 and Hsp27^8–12^. Over-expression of Hsp70 and Hsp27 is thought to lower the efficacy of Hsp90 inhibitors as therapeutics^13–18^. PU-H71, a purine-based Hsp90 inhibitor, has shown improved preclinical and clinal efficacy and is a promising application as a theragnostic platform for precision medicine targeting the epichaperome^7,19^. Furthermore, preclinical and clinical data indicate that 4-(1*H*-pyrazolo[3,4-*b*]pyridine-1-yl)benzamide TAS-116 (Pimitespib), a selective oral inhibitor of cytosolic Hsp90α and β, exhibits a potent anti-tumor activity in various cancers with minimal side effects, including visual disturbances^20–23^. Pimitespib has recently been approved for clinical use in Japan. However, to date, no Hsp90 inhibitor is approved by the FDA, and the full clinical potential of Hsp90 inhibition for treating cancers remains to be harnessed.

To this end, alternative strategies of Hsp90 inhibition are being pursued to improve efficacy, including targeting the Hsp90 middle domain, which binds client proteins, and the C-terminal ATP binding domain^24^. For example, Novobiocin, a C-terminal ATP binding inhibitor^25^, is currently being used as a chemical scaffold to develop more specific and potent inhibitors of Hsp90^1,26^. Interestingly, Hsp90 C-terminal inhibitors do not induce the HSR, and NCT-58 triggers the death of rapidly proliferating and stem-like cells of trastuzumab-resistant HER2-positive breast cancer models through down-regulation of HER2 and Heat Shock Factor 1 (HSF1). Alternatively, inactivation of Hsp90 could be achieved through inhibition of specific co-chaperone interactions or post-translational modifications of Hsp90^1,27^. Attempts to abrogate the HSR by developing inhibitors of Hsp27, Hsp70, and the HSF1 transcription factor are also being pursued^28–30^.

Capsaicin (8-methyl-N-vanillyl-6-nonenamide) is the primary pungent ingredient of hot peppers of the genus *Capsicum*. Capsaicin is an agonist of transient receptor potential vanilloid-1 (TRPV1) that triggers both the sensations of burning and pain, but also promotes the desensitization of peripheral nerves to noxious stimuli^31^. Capsaicin is approved by the FDA for topical use in pain management of arthritis, diabetic neuropathy, and neuralgia and also to treat post-surgical neuropathic pain in cancer patients^32^. Numerous reports have shown that capsaicin induces cell cycle arrest and apoptosis in cancer cell lines^33^ and autophagy in anaplastic thyroid carcinoma cells^34^. Interestingly, co-treatment of cancer cells with capsaicin and capsazepine (a TRPV1 antagonist) showed that cells still die by apoptosis^35^, suggesting that cytotoxicity was independent of TRPV1 activation, which was confirmed in an oral cancer model^36^. Several mechanisms of action have been proposed for the cytotoxic properties of capsaicin, including NF-κB inactivation^37^, estrogen receptor (ER) stress-mediated apoptosis^38^, generation of reactive oxygen species (ROS), disruption of the mitochondrial transmembrane potential^39^, inactivation of the EGFR/HER-2 pathway^40^, cyclin-dependent kinase blockade^41^, anti-angiogenic activity by direct inhibition of Src kinase^42^ and the VEFG pathway^43^, and inactivation of the E2F-Rb pathway^44^, among others.

Our data suggest that the cytotoxic effects of capsaicin in cancer cells may also involve the inactivation of Hsp90, and most of the inactivated pathways above are driven by protein clients of Hsp90. We have previously shown that capsaicin treatment destabilizes several Hsp90 client proteins^45^. In this report, we demonstrate that Hsp90 is a critical target of capsaicin in cancer cells. Specifically, we show that capsaicin binds to the N-terminal domain of Hsp90 in a manner unique from classical Hsp90 N-terminus inhibitors, and rather than inducing Hsp70 and the HSR, capsaicin triggers the lysosomal degradation of Hsp70 in various cancer cell lines. Remarkably, capsaicin treatment did not affect Hsc70, which shares ~ 85% amino acid sequence identity with Hsp70^46^. Further, low concentrations of doses of capsaicin enhanced 17-AAG tumor-cell killing using two-dimensional and spheroid culture systems. These data support the use of capsaicin as a scaffold to develop novel N-terminal Hsp90 inhibitors that circumvent over-expression of the Hsp70 protein and/or use it as a co-therapeutic.

## Results

### Hsp90 is a target of capsaicin in cancer cells

CAP-OH was prepared by etherification of phenolic hydroxyl of capsaicin with t-BOC-protected tetra-ethylene glycol amine, followed by trifluoroacetic acid deprotection of the amino group. CAP-NH_2_ was made by acylation of vanillylamine with 10-azidodecanoyl chloride with subsequent reduction of the azido functionality (see “Methods”). The two capsaicin derivatives were immobilized on Sepharose resin using divinylsulfone chemistry as previously described^47^. This resin was used in pulldown experiments to identify proteins that bind capsaicin in MCF7 cells.

**Figure 1:**
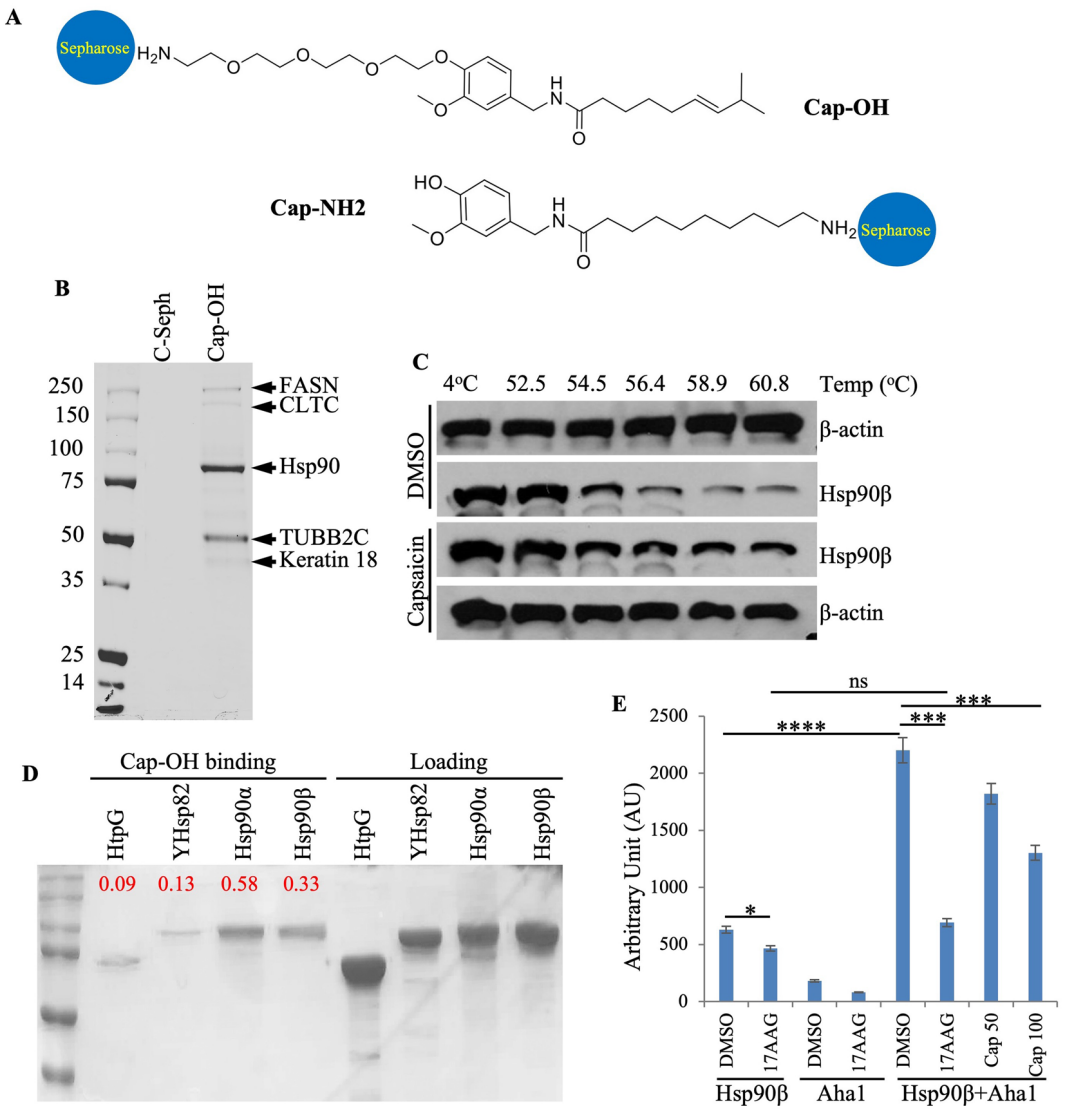
Capsaicin binds to Hsp90 ex vivo and in cells. (**A**) Schematic representation of capsaicin immobilized on Sepharose resin through OH (Cap-OH) or NH2 (Cap-NH2) groups as described in “Methods”. (**B**) Coomassie blue staining of proteins from MCF7 cell extracts bound to capsaicin immobilized on Cap-OH resin. Indicated proteins were identified by mass spectrometry. Hsp90 α and β are the major capsaicin interactors but other proteins such as FASN (fatty acid synthase) and TUBB2C (tubulin beta-2C chain + alpha-4A chain) were identified. Sepharose resin without capsaicin was used as a control. (**C**) Cellular thermal shift assay using Hs578T human triple-negative breast cancer cell line. (**D**) Pull-down of purified Hsp90 orthologs using Cap-OH Sepharose resin. HTPG: bacterial Hsp90; YHsp90: yeast Hsp82 and human cytosolic Hsp90α and β. The numbers in red indicate the ratios of binding relative to the corresponding loading/input**.** (**E**) 50 and 100 μM capsaicin inhibit the Hsp90β ATPase activity stimulated by Aha1. 10 μM 17-AAG is used as a positive control. The experiment was repeated three times. Analysis of variance and Bonferroni tests were performed to test for the statistical significance of values. *P*-value*:* **P < 0.01, ***P < 0.001, ****P < 0.000.

Cell lysates from human breast cancer MCF7 cells were incubated with Cap-OH (Fig. 1A) or control resins overnight at 4 °C. Bound proteins were separated by SDS-PAGE and stained with Coomassie blue. Visibly stained bands were then identified by mass spectrometry. As shown in Fig. 1B, cytosolic Hsp90α (399 peptides) and β (112 peptides) were pulled down by Cap-OH resin, indicating that Hsp90 and capsaicin interact. In the 90 kDa molecular weight band, the importin subunit beta-1 (KPNB1) (59 peptides) and isoform 1 of Transportin-1 (TNPO1) (17 peptides) were also identified. Two additional substantial proteins at different molecular weights were identified: Fatty acid synthase (FASN) (250 kDa) and Tubulin beta-2C chain + alpha-4A chain TUBB2C (50 kDa). We also identified minor traces of the clathrin heavy chain (CLTC). However, it remains undetermined if these proteins interact directly with capsaicin or if they are pulled down as components of the Hsp90 complexes. Consistent with the latter possibility, tubulin^48,49^ and FASN^50^ have been shown to interact with Hsp90, and importin β1 forms a complex with Hsp90 to facilitate the nuclear trafficking of nuclear receptors and telomerase reverse transcriptase (hTERT)^51,52^.

To validate Hsp90 as a cellular target of capsaicin, we used the cellular thermal shift assay (CETSA) to evaluate drug target engagement in cells^53^. Human Hs578T breast cancer cells were treated with 200 µM capsaicin for 1 h. Cell protein extracts were heated in a thermocycler using a temperature gradient from 52.5 to 68.8 °C to cause protein melting and denaturation. Cell extracts were then centrifuged to remove precipitated proteins. If Hsp90 is a target of capsaicin in cells, binding would promote Hsp90 stability and resistance to temperature-induced denaturation. As shown in Fig. 1C, treatment with capsaicin attenuated Hsp90β loss, indicating that capsaicin binds to Hsp90 in Hs578T cells.

To test capsaicin selectivity and direct binding to Hsp90, Cap-OH resin was used to pull down purified human cytosolic Hsp90α and β as well as the yeast (Hsp82) and bacterial (HtpG) orthologs. HtpG was expressed in *E. coli* but yeast and human proteins were expressed in insect sf9 cells. As shown in Fig. 1D, minimal to no binding was observed using HtpG and yeast hsp82, but both human Hsp90α and β bind the Cap-OH resin.

To determine the functional impact of capsaicin on Hsp90 in vitro, we tested the ability of capsaicin to inhibit the Aha1-stimulated ATPase activity of Hsp90. As shown in Fig. 1E, capsaicin reduced the ATPase activity of Hsp90, albeit to a lesser extent than the classical Hsp90 inhibitor 17AAG; capsaicin (100 µM) only inhibited about 50% of the ATPase activity. Importantly, as shown below, this level of inhibition correlates with the overall efficacy of capsaicin in terms of cell killing, hsp90 client destabilization^45^, and reduction of Hsp70 protein levels.

**Figure 2:**
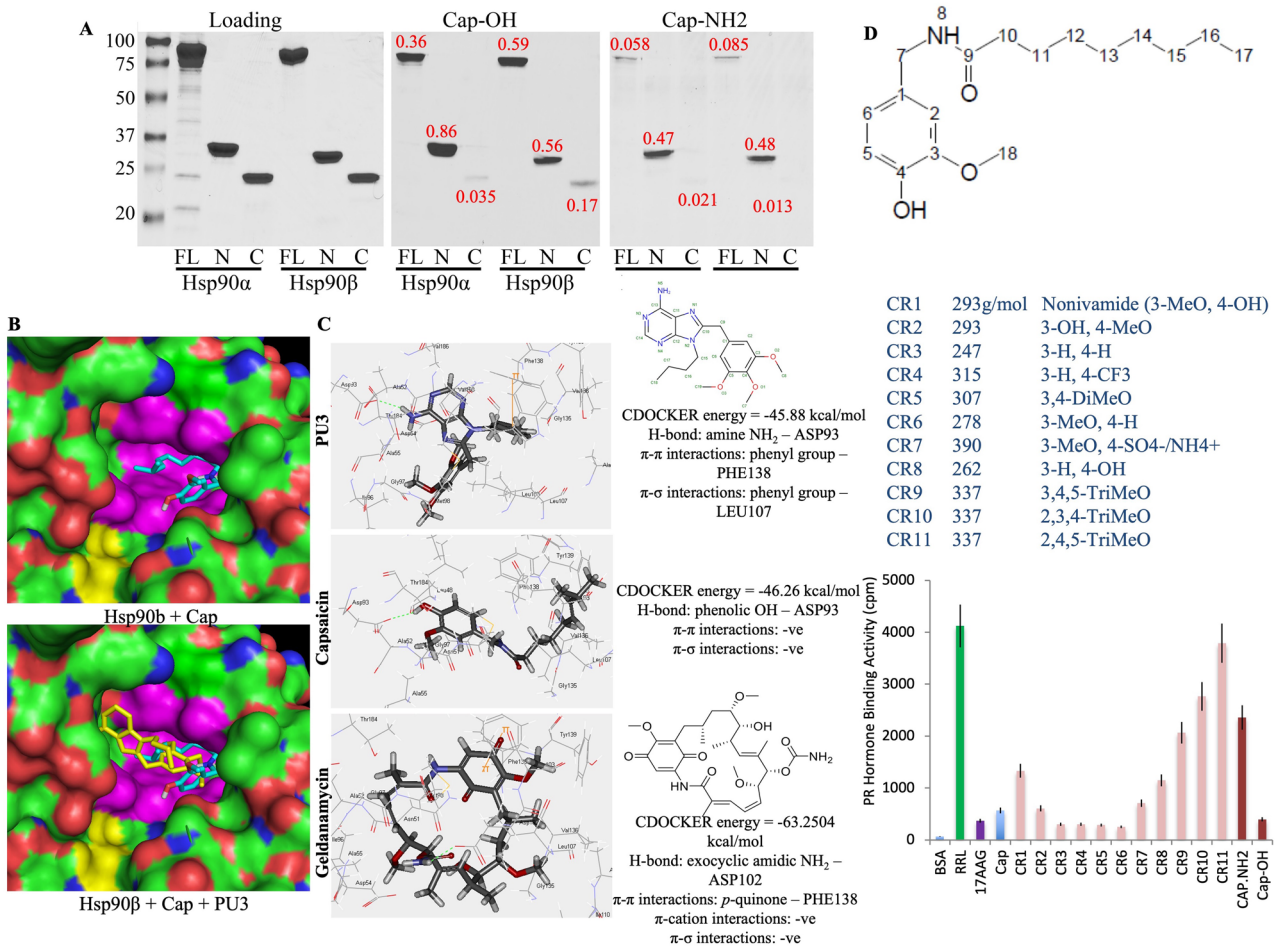
Capsaicin binds to the N-terminal ATP binding domain of Hsp90. (**A**) SDS-PAGE and Coomassie blue staining of Hsp90 full length (FL), N-terminal (N, residues 1–212), and C-terminal (C, 616-C-terminal) domains bound to Cap-NH2 and Cap-OH. Loading indicates 5 μg of protein. The numbers in red indicate the ratios of binding relative to the corresponding loading/input**.** The faint bands of lower molecular weight seen in Hsp90 full length are degradation products. (**B**) Molecular docking confirming the binding of capsaicin to the N-terminal domain of Hsp90 alone (top panel) or with the purine-based inhibitor, PU3 (PDB 10.2210/pdb1UYM/pdb). (**C**) Molecular docking of capsaicin compared to the binding of PU3 and geldanamycin with amino acid residues potentially involved in the interaction. (**D**) Impact of various capsaicin derivatives (100 µM) on the progesterone reconstitution complex and its ability to bind ^3^H-progesterone. The PR reconstitution was performed using reticulocyte lysate as a source of chaperones. 10 µM 17-AAG was used as a positive control. Cpm: count per minute. The experiment was repeated three times. *Error bars* represent means ± S.D. Unpaired two-tailed *t*-test was used for significance.

To delineate the capsaicin binding site on Hsp90, we used capsaicin resins to pull down full-length hsp90α and β as well as the N-terminal and C-terminal domains (Fig. 2A). Interestingly, the C-terminal fragment (616-Ct) showed no significant binding, but the N-terminal ATP-binding domain (amino acid residues 1–212) was efficiently pulled down by the Cap-OH resin. This was confirmed by analysis via in silico molecular docking, which predicted capsaicin binding to the ATP site of the N-terminal domain (Fig. 2B). These predicted docking interactions were unique from those for the Hsp90 inhibitor PU3. Interestingly, both capsaicin and PU3 formed hydrogen bonds with Asp93 of Hsp90, but PU3 was also predicted to interact with Phe138 and Leu107 (Fig. 2C).

Docking studies also suggested that flexibility of the aliphatic chain of capsaicin is important for Hsp90 binding. Consistent with this, immobilization of capsaicin via the aliphatic side chain to generate Cap-NH2 resin (Fig. 1A) dramatically reduced Hsp90 binding compared to capsaicin immobilization through the C_3_-OH on the vanilloid ring (Fig. 2A). These results agree with the in silico-estimated affinities of − 62.8 kcal/mol and − 76.38 kcal/mol for Cap-NH2 and Cap-OH, respectively (Fig. 2C). Furthermore, analysis using derivatives of capsaicin^54,55^ confirmed that binding with the vanilloid ring could tolerate substantial chemical modifications (Fig. 2D). However, the incorporation of multiple 3 methoxy groups in positions 2, 3, 4 and 5 significantly reduced the ability of capsaicin to inhibit progesterone receptor (PR) reconstitution, which was used as a biomarker for Hsp90-mediated client protein folding, indicating that these bulky modifications are unfavorable for the Hsp90 binding.

### Capsaicin induces lysosomal (or autophagic) degradation of Hsp70

**Figure 3:**
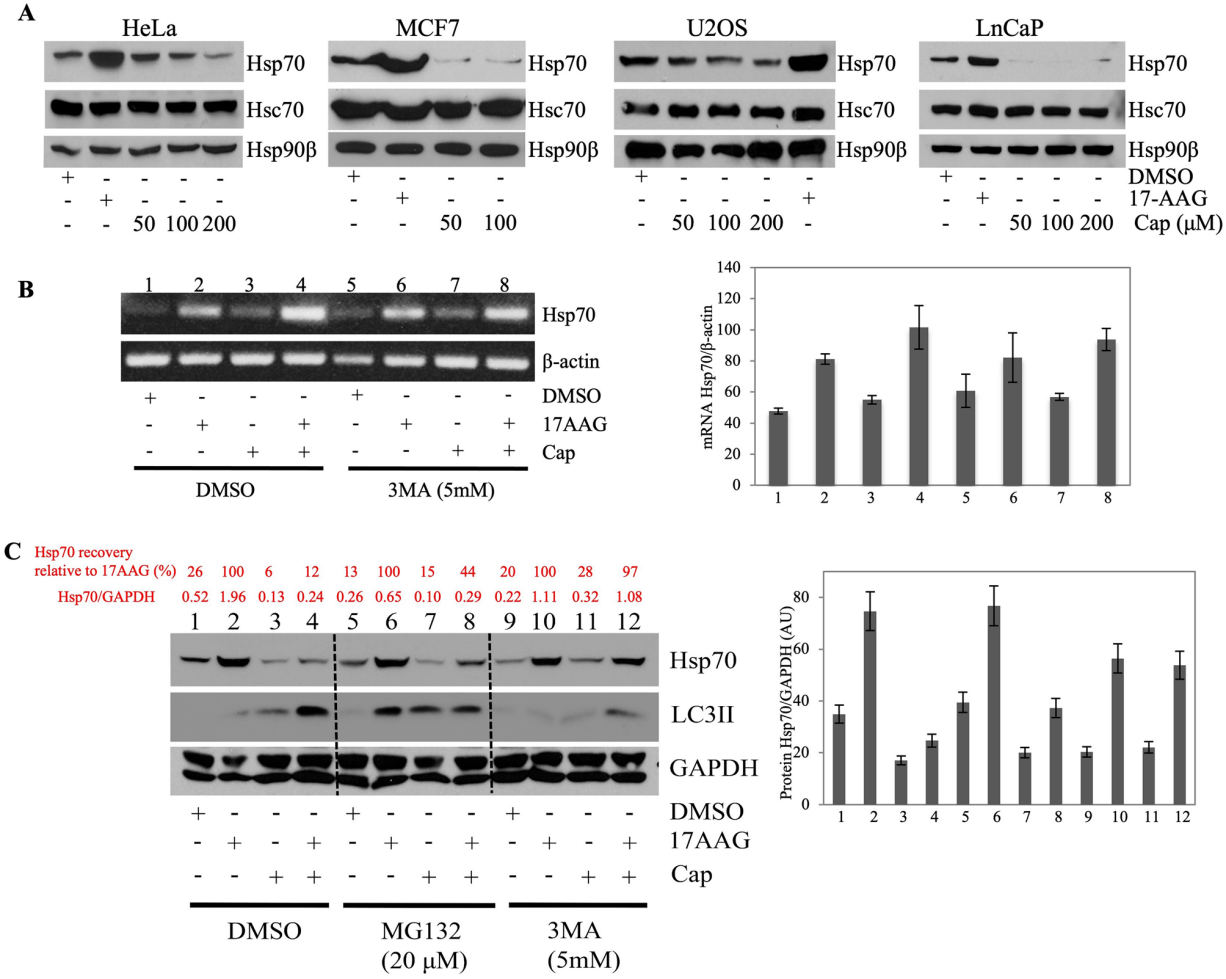
Capsaicin induces Hsp70 protein lysosomal degradation. (**A**) HeLa PR_B_, (not sure we have defined what HeLA PRb means) MCF7, U2OS, and LNCaP cell lines were treated with 50, 100, and 200 μM capsaicin for 24 h. Cell lysates were prepared and Western blotted for Hsp70, Hsc70, and Hsp90β. DMSO was used as a negative control; 17-AAG (0.2 μM) was used as a positive control; and β-actin was used as a loading control. For each cell line, the experiment was repeated three times. (**B**) RT-PCR analysis of MCF7 cells treated with either capsaicin (200 μM) or 17-AAG (200 nM) or in combination in the presence or absence of 5 mM 3MA. DMSO was used as a negative control. Quantification of Hsp70 band intensity relative to β-actin loading control is shown in the right panel**.** (**C**) Western blot analysis of cell lysates from B in addition to cells treated with 20 μM MG132. Cells were treated with 3MA or MG132 for 6 h. Quantification of Hsp70 band intensity relative to GAPDH control is shown in the right panel. The recovery rate of Hsp70 relative to 17-AAG upon treatment with MG132 or 3MA was determined by dividing Hsp70/GAPDH rate over the Hsp70 rate induced by 17-AAG.

Capsaicin treatment had a distinct impact on cancer cells when compared to 17-AAG. Our previous studies showed that, like 17-AAG, capsaicin reduced the cellular levels of Hsp90 client proteins including the steroid receptors (androgen receptor (AR), glucocorticoid receptor (GR), and progesterone receptor (PR)), and kinases (CDK4, ChK1, Raf1, ILK, and HER2)^45^. However, increased expression of HSR biomarkers, including Hsp70, was not observed in HeLa, MCF7, U2OS, or LnCaP human cancer cell lines. Rather, the level of Hsp70 protein was reduced after capsaicin treatment in all cell lines tested (Fig. 3A). However, the protein level of Hsc70 showed no change upon capsaicin treatment (Fig. 3A). Hsp70 and Hsc70 share ~ 85% amino acid sequence identity^56^, indicating that capsaicin selectively affected Hsp70. Together, these data indicate that capsaicin inhibits Hsp90 chaperoning by binding to the N-terminus ATP binding site and does not trigger the HSR response typified by Hsp70 induction as observed with classical N-terminus inhibitors^45^.

Capsaicin-mediated down-regulation of Hsp70 (Fig. 3A) could result from repression of Hsp70 gene transcription, translation, or destabilization of Hsp70 protein. The level of Hsp70 mRNA was increased after capsaicin treatment (Fig. 3B, lanes 1 and 3), while the protein level was decreased (Fig. 3C, lanes 1 and 3). However, the increase in Hsp70 mRNA caused by capsaicin treatment was minimal compared to the marked up-regulation caused by 17-AAG (Fig. 3B, lanes 1 and 2), which was further induced when cells were treated with capsaicin and 17-AAG (Fig. 3A, lane 4). Consistent with the effect of capsaicin on Hsp70 protein, induction of Hsp70 by 17-AAG was drastically attenuated by capsaicin co-treatment (Fig. 3C, lanes 2 and 4).

**Figure 4:**
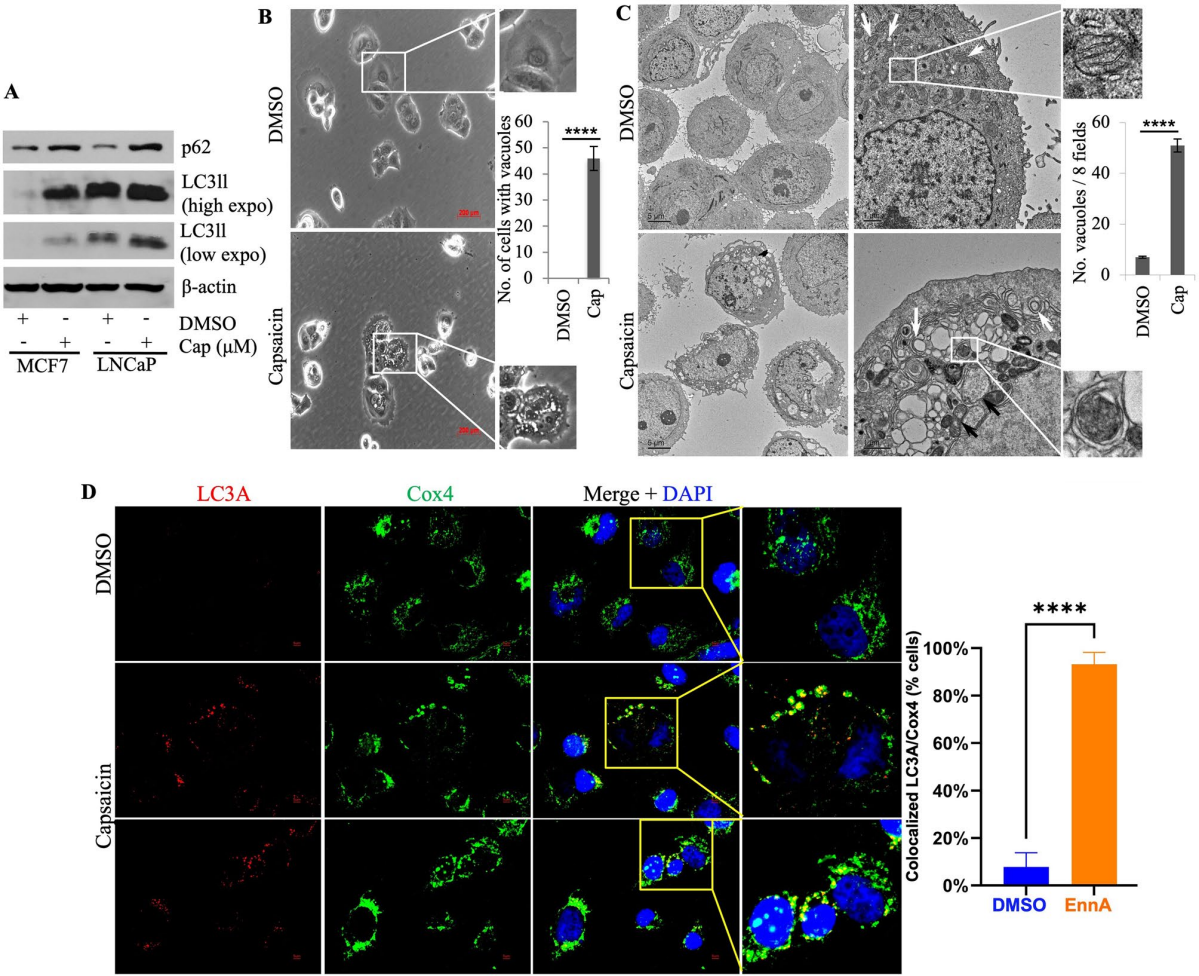
Capsaicin induces mitophagy in MCF7 and LNCaP cells. (**A**) MCF7 and LNCaP cells were treated with 200 μM capsaicin for 24 h; cell lysates were prepared and Western blotted for p62/SQSTM1 and LC3II. β-actin was used as a loading control (left panel). This experiment was performed at least twice. (**B**) Representative images of light microscopic analysis of MCF7 cells treated with 200 μM capsaicin for 12 h showed numerous vacuoles. Insets: a single cell with no vacuoles in the DMSO group and a single cell full of vacuoles in the capsaicin group. Quantification of 200 vacuolated cells from three biological replicates of either DMSO or capsaicin treatment groups is shown on the right. The scale bar represents 200 μm. (**C**) Transmission electron microscopic analysis of MCF7 cells treated with 200 µM capsaicin for 12 h showed a marked accumulation of double-membrane vesicles. Arrows point to healthy mitochondria with intact cristae in the DMSO group and swollen mitochondria with lost cristae in the capsaicin group. Insets: a healthy mitochondrion in the DMSO group and a diseased mitochondrion engulfed by a cup-shaped double membrane structure in the capsaicin group. Quantification of vacuoles larger than 250 nm in eight different fields, either in the DMSO or capsaicin treatment groups, is shown on the right. The scale bars represent 5 μm (left panels) and 1 μm (right panels). (**D**) Immunocytochemistry analysis of Hs578T cells treated with 200 µM capsaicin or DMSO control for 12 h. Coverslips were immunostained with LC3A antibody as an autophagy marker and Cox4 as a mitochondria marker. The left panel is the average of the number of cells showing colocalization of LC3A and Cox4 (yellow color) from three controls and three treated samples. 100 cells per sample were counted and plotted using ImageJ Version 1.54e and PRISM software. *P*-value*:* ****P < 0.0001.

The roles of the proteasomal and lysosomal degradation pathways in mediating capsaicin-induced Hsp70 degradation were then studied. MCF7 cells were treated with capsaicin in the presence of a proteasome inhibitor (MG132) or autophagy inhibitor (3MA). As shown in Fig. 3C (lanes 5 and 7), 6 h treatment with MG132 did prevent about 16% and 44% of Hsp70 degradation with capsaicin treatment, alone or in combination with 17-AAG, respectively (Fig. 3C lanes 6 and 8). However, treatment with 3MA promoted almost full recovery (97%) of Hsp70 induced by 17-AAG (Fig. 3C lanes 10 and 12). Of note, the profile of Hsp70 mRNA was consistent with and without 3MA treatment (Fig. 3B). Accumulation of LC3II indicated that both 17-AAG and capsaicin-induced autophagy in MCF7 cells (Fig. 3C lane 3). Interestingly, a significantly higher accumulation of LC3II occurred when capsaicin was combined with 17-AAG compared to capsaicin alone (Fig. 3C lanes 3 and 4), suggesting that capsaicin-mediated lysosomal degradation was further accentuated by 17-AAG, which correlated with the drastic loss of Hsp70 protein level in the capsaicin-17-AAG co-treatment group (Fig. 3C lanes 2 and 4). As expected, 3MA reduced LC3II accumulation (Fig. 3C lanes 9–12), and together, these data suggest that co-treatment of capsaicin and 17-AAG stimulates autophagy, which promotes Hsp70 degradation. Autophagy induction (Fig. 4A) and Hsp70 degradation (Fig. 3A) were also observed in LNCaP cells treated with capsaicin.

Consistent with the paradigm above, light microscopic analysis of MCF7 cells showed that capsaicin-treated cells developed vacuoles (Fig. 4B), which were further resolved using transmission electron microscopy as double-membrane lining structures, indicative of autophagosomes, (Fig. 4C). Interestingly, the mitochondria in capsaicin-treated cells were also deformed, exhibiting irregular membranes (Fig. 4C). Specifically, the normal pattern of mitochondrial cristae (inner mitochondrial membrane) was lost, and unhealthy mitochondria were present in autophagosome structures, suggesting active mitophagy as well (Fig. 4C). This was further confirmed using immunocytochemistry analysis showing a reduction in staining by Cox4, a mitochondrial marker, and co-localization of Cox4 with LC3A puncta in the capsaicin-treated cells (Fig. 4D). These findings agree with two reports showing that capsaicin treatment induces macro-autophagy in MCF7 breast cancer cells^57^ and that dihydrocapsaicin (a saturated analog of capsaicin) induces mito-autophagy in HCT 116 colon cancer cells^58^.

### Capsaicin prevented 17AAG-induced over-expression of Hsp70 and improved 17-AAG anti-tumor activity

**Figure 5:**
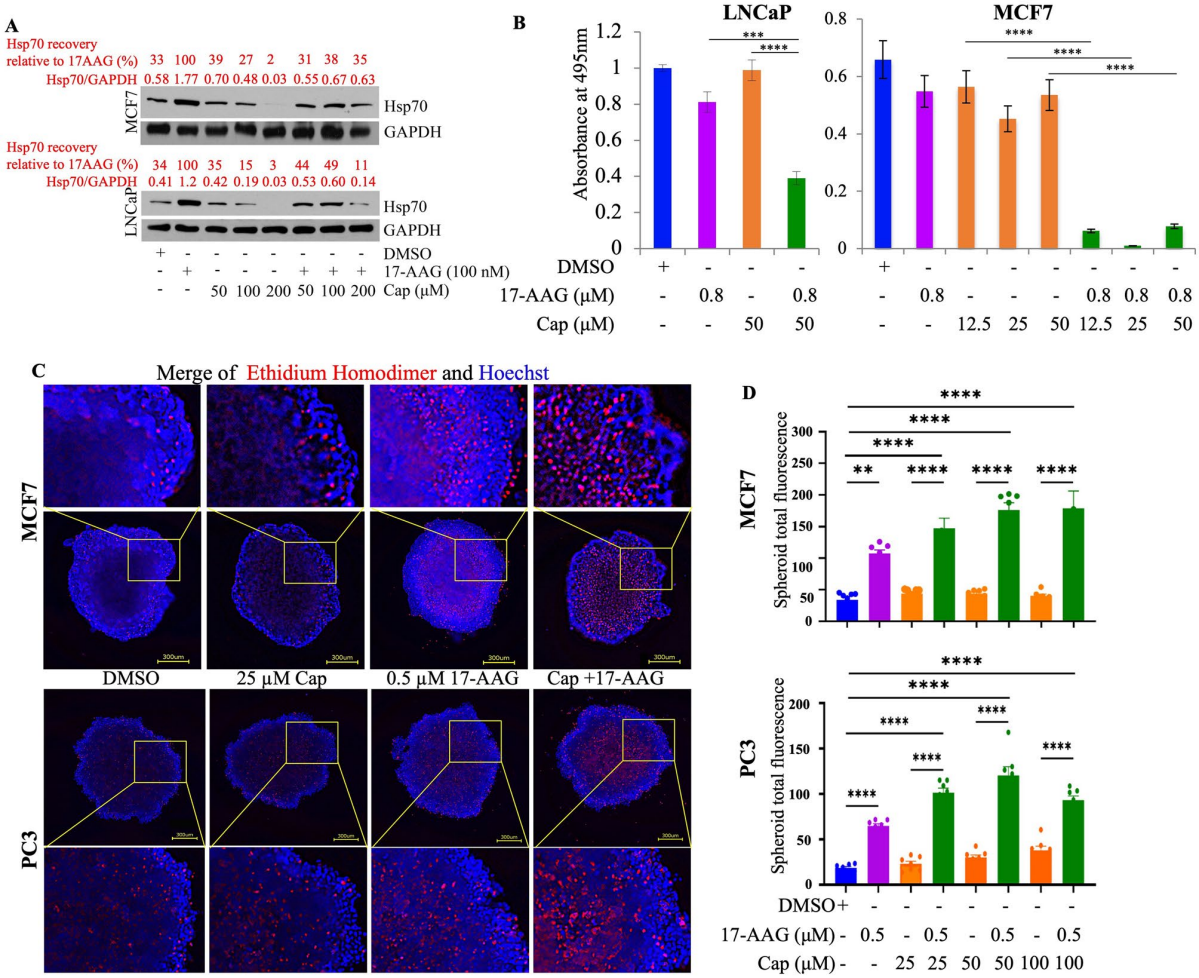
Capsaicin improves 17-AAG cytotoxicity for breast and prostate cancer lines. (**A**) MCF7 and LNCaP cells were treated with 50, 100, and 200 μM capsaicin, either alone or in combination with 100 nM 17-AAG for 24 h. Cell lysates were prepared and Western blotted for Hsp70. Capsaicin prevented 17-AAG-induced up-regulation of Hsp70 in both cell lines. DMSO was used as a negative control. GAPDH was used as a loading control. (**B**) MTT cell survival assays of LNCaP cells treated for 60 h with either 17-AAG (800 nM) alone or in combination with various concentrations of capsaicin. DMSO was used as a negative control. (**C**,**D**) Combination with capsaicin improves the efficiency of 17-AAG to kill tridimensional spheroid growth of breast MCF7 and prostate PC cancer models. The standard deviation of quadruplet samples is shown as error bars. The experiment was repeated three times. Analysis of variance and Bonferroni tests were performed to test for the statistical significance of values. *P*-value: **P < 0.01, ***P < 0.001, ****P < 0.0001.

Pharmacological inhibition of Hsp90 by N-terminal inhibitors, including 17-AAG, induces the HSR, which is thought to reduce the efficacy of Hsp90 inhibitors through up-regulation of the anti-apoptotic proteins Hsp70 and Hsp27^59,60^. We therefore further tested whether capsaicin-dependent inhibition of 17-AAG-induced Hsp70 expression would affect tumor cell killing. MCF7 and LNCaP cells were treated with 50, 100, and 200 μM capsaicin with and without 100 nM 17-AAG. In a concentration-dependent manner, capsaicin abolished the 17-AAG-induced over-expression of Hsp70 in the co-treated group compared to 17-AAG alone (Fig. 5A, Quantification in Supplementary data). Indeed, in the presence of 17-AAG, 200 µM capsaicin reduced the Hsp70 level to that of DMSO control; 35% and 33% respectively compared to 17-AAG alone. The impact of capsaicin is even more in LNCaP where Hsp70 is reduced to 1/3 of the level of control DMSO; 11% and 34% respectively compared to 17-AAG alone.

As shown in Fig. 5B, the viability of LNCaP cells treated for 48 h with capsaicin (50 µM) and 17-AAG (800 nM) was markedly lower than for either capsaicin or 17-AAG alone. This result was further demonstrated using MCF7 cells where enhanced killing occurred at concentrations of 12.5, 25, and 50 µM capsaicin combined with 17-AAG (800 nM); Fig. 5B). A similar effect of capsaicin was observed using a tridimensional (3D) spheroid growth assay with MCF7 and the prostate cancer cell line PC3. As shown in Fig. 5C,D, the combination of capsaicin and 17-AAG produced significantly greater cell killing of MCF7 and PC3 3D-spheroids as indicated by Ethidium Homodimer-1 (EthD-1) staining (Fig. 5D), a marker for dead cells.

## Discussion

Capsaicin has been shown to trigger a variety of signaling events in cancer cell lines. The mechanism by which capsaicin induces apoptosis in cancer cells is not well understood, but it appears to be independent of the TRPV1 receptor as neither capsazepine, a TRPV1 antagonist, nor intracellular Ca^2+^ chelators have been found to inhibit apoptosis^35,36,61,62^. In contrast, Reilly and coworkers have shown that TRPV1 may play a more dominant role in capsaicinoid toxicity in non-cancerous lung cells, and some role in human lung adenocarcinoma (A549) cells, which were more sensitive to the cytotoxic effect of capsaicin than human hepatocarcinoma (HepG2) cells, which expressed less TRPV1 receptor^55,63^. Regardless, capsaicin can block the activity of many oncogenic signaling proteins including NF-κB, ER, EGFR/HER2, CDK4, Src, VEGF, and PI3K/Akt, among others. Thus, there exists considerable pleiotropy with regard to the mechanism of capsaicin-induced cancer cell death^64^. Importantly, however, many of the signaling proteins inactivated by capsaicin are clients of Hsp90, which is consistent with capsaicin binding and inactivating Hsp90 as shown in this report. Hence, it is reasonable to propose that the seemingly pleiotropic effects of capsaicin may, in part, result from the inhibition of Hsp90 in cancer cells.

This hypothesis is supported by our previous report showing that treatment of several cancer cell lines with capsaicin resulted in dose-dependent degradation of Hsp90 client proteins, including the steroid receptors AR, PR, and GR, and HER2, Akt, Raf, CDK4, and Chk1 kinases^45^. Pulldown experiments coupled with mass spectrometry analysis strongly suggest that cytosolic Hsp90α and β are crucial cellular targets of capsaicin and that capsaicin interacts with the N-terminus ATP binding domain of Hsp90 to promote cancer cell death through autophagy. This domain is the target of Hsp90 inhibitors including geldanamycin and its derivatives, 17-AAG, resorcinol compounds, and ATP-based structures such as PU and others. The apo N-terminal domain of Hsp90 has a highly flexible lid segment formed by residues 107 to 141^65^ and can be significantly affected by ligand binding^66^. Structural comparison of N-terminal domain interaction with ligands showed that the main structural variations are limited to the conformation of residues 104 to 111 located in the α-helix 3 domain^67^. This region can adopt a helical or more open conformation in yeast Hsp90, but it is structured as an extended loop that closes the binding site of human Hsp90^68^. The key residues for ligand binding in all Hsp90 orthologs are centered around residue Asp93. These include Asn51, Ser52 (Ala38 in yeast), Ala55, Met98, and Thr184. These structural changes are potentially important to the selective binding of capsaicin to Hsp90α and β compared to yeast and bacterial Hsp90. However, more structural studies are needed to better define the molecular motifs underlying this selectivity. It remains intriguing and potentially significant to understand how capsaicin binding the N-terminus of Hsp90 does not induce the HSR akin to classical N-terminal binding inhibitors of Hsp90. Indeed, It is well known that 17-AAG and other Hsp90 ATP competitors induce HSF1^69^ through dissociation of the Hsp90:HSF1 complex and prolongation of Hsp70 promoter activation. However, cell treatment with capsaicin selectively down-regulated Hsp70 protein in various cancer models, including HeLa, MCF7, U2OS, and LNCaP cell lines (Fig. 3A). Interestingly, the combination of capsaicin and 17-AAG amplified the activation of HSF1 as indicated by the large accumulation of Hsp70 mRNA. However, the Hsp70 protein was efficiently degraded, which is predicted to deprive cells of the pro-survival activities of Hsp70, including inhibition of apoptosis^11^.

The ability of capsaicin to selectively reduce Hsp70, but not Hsc70, was also intriguing. The two isoforms share more than ~ 85% amino acid sequence identity, and their overall structures are remarkably similar. However, some structural differences do occur in their carboxy-terminal domains (amino acids 510–641), which show only 70.5% amino acid identity. These differences between Hsp70 and Hsc70 could impact their activities. For example, Menoret and coworkers measured the peptide-binding affinities of purified Hsp70 and Hsc70 using three different radiolabeled peptides under a variety of buffer conditions in vitro^70^. They found that Hsp70 binds to peptides with higher affinity than Hsc70 does. Whether these differences play a role in the capsaicin-induced selective degradation of Hsp70 remains to be determined. It is also possible that this selectivity between the two isoforms is regulated by differences in the complex pattern of chaperones' posttranslational modifications, known as chaperone code, including phosphorylation, acetylation, ubiquitination, AMPylation, and ADP-ribosylation^71,72^.

Our data show that capsaicin treatment induced autophagy and mitophagy in MCF7 cells (Fig. 4C). In capsaicin-treated cells, mitochondrial cristae were visibly compromised. The mitochondrial cristae constitute the inner mitochondrial membrane, which harbors protein complexes I–V of the electron transport system (ETS). Using BxPC-3 and AsPC-1 (pancreatic cancer) cell lines, Srivastava and coworkers showed that capsaicin blocked enzymatic activity and reduced protein levels of complexes I and III, thus disrupting the mitochondrial ETS. This resulted in increased production of reactive oxygen species (ROS), which subsequently disrupted mitochondrial transmembrane potential, leading to apoptosis^73^. Several other reports have also documented that capsaicin disrupted mitochondrial function and induced ROS production in cancer cells, leading to death^39,74–79^. Importantly, Hsp90 impacts mitochondrial ETS function, and inhibition of Hsp90 causes mitochondrial dysfunction^80^.

How Hsp70 is degraded in a lysosomal-dependent manner, as indicated by the rescue of Hsp70 with 3MA, remains unknown. Interestingly, over-expression of Hsp70 prevented starvation-induced autophagy by up-regulation of the mTOR-Akt pathway^81^. George and coworkers showed that inhibition of Hsp70 by the small molecule compound PES resulted in marked induction of autophagy^82^. These data seem to indicate that Hsp70 is necessary for the suppression of autophagy under normal physiological conditions. Hsp70, however, is not the only protein that suppresses autophagy, as *Hsp70.1.3*^−/−^ mouse embryonic fibroblasts (MEFs) maintain basal levels of autophagy^83^. It is important to study how capsaicin induces autophagy and to further test how this leads to the degradation of Hsp70 without the degradation of the classical autophagic flux p62/SQSTM1 (Fig. 4A).

Hsp70 is a major anti-apoptotic protein known to be up-regulated in many cancers^84–87^. 17-AAG and other N-terminal Hsp90 inhibitors cause over-expression of Hsp70 as a side effect. Our data show that co-treatment of capsaicin with 17-AAG prevented the 17-AAG-mediated up-regulation of Hsp70, which translated into more potent cytotoxicity and killing of multiple cancer cell types using both standard and 3D culture models. These findings suggest that capsaicin could potentially be used in combination with N-terminal Hsp90 inhibitors to induce a synergistic anti-tumor effect.

## Methods

### General methods for capsaicin derivative synthesis

Organic solvents were dried and distilled before use; tetrahydrofuran was distilled from sodium/benzophenone ketyl. Other reagents were obtained from Aldrich or VWR and used as received, unless otherwise noted. Flash chromatography was performed using 40–63 µm silica gel. All NMR spectra were recorded in CDCl_3_ using a 400 MHz instrument.

#### Synthesis of CAP-OH


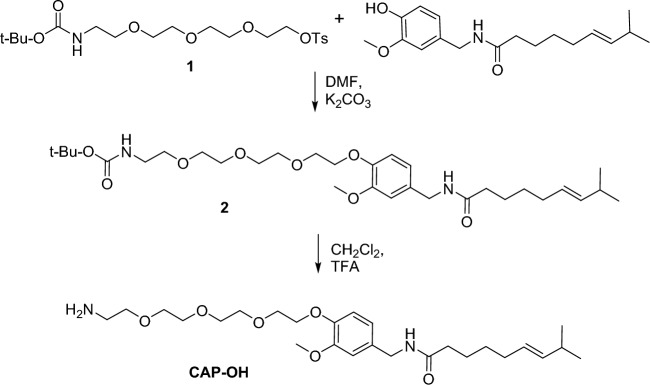


##### *tert*-Butyl (E)-(2-(2-(2-(2-(2-methoxy-4-((8-methylnon-6-enamido)methyl)phenoxy) ethoxy)ethoxy)ethoxy)ethyl)carbamate (2)

2,2-dimethyl-4-oxo-3,8,11,14-tetraoxa-5-azahexadecan-16-yl 4-methyl benzenesulfonate (**1**)^1^ (0.915 g, 2.05 mmol) and potassium carbonate (0.181 g, 1.31 mmol) were added to a solution of capsaicin (0.400 g, 1.31 mmol) in DMF (13 mL). The reaction mixture was stirred at 75 °C overnight, cooled down to room temperature and extracted with diethyl ether (250 mL). The organic layer was washed with water (5 × 50 mL), brine (100 mL), and dried over MgSO_4_. The solvent was evaporated in a vacuum and the crude product was purified by chromatography (silica gel/ethyl acetate) to give 624 mg (80%) of capsaicin derivative **2** as a white amorphous solid.

^1^H-NMR: 6.85–6.87 (m, 1H), 6.77–6.80 (m, 2H), 5.83 (s, 1H), 5.04 (s, 1H), 4.36–4.37 (d, J = 5.6 Hz, 2H), 4.16–4.18 (t, 2H), 3.86–3.89 (t, 2H), 3.83 (s, 3H), 3.73–3.75 (m, 2H), 3.60–3.68 (m, 6H), 3.50–3.53 (t, 2H), 3.26–3.28 (m, 2H), 2.18–2.22 (t, 2H), 1.63–1.67 (m, 2H), 1.43 (s, 9H), 1.36–1.29 (m, 12H), 0.85–0.89 (t, 3H).

^13^C-NMR: 173.12, 156.19, 149.98, 147.89, 131.99, 120.26, 114.09, 111.96, 79.35, 71.04, 70.82, 70.75, 70.45, 70.38, 69.84, 68.90, 56.12, 43.56, 40.55, 37.04, 32.00, 29.53, 29.52, 29.35, 28.62, 26.00, 22.83, 14.28.

##### (*E*)-*N*-(4-(2-(2-(2-(2-aminoethoxy)ethoxy)ethoxy)ethoxy)-3-methoxybenzyl)-8-methylnon-6-enamide (CAP-OH)

Trifluoroacetic acid (409 mg, 3.6 mmol) was added to a solution of the capsaicin derivative (**2**, 510 mg, 0.9 mmol) in DCM (5 mL) at room temperature. The reaction mixture was stirred overnight, quenched with saturated sodium bicarbonate (50 mL), and extracted with DCM (200 mL). The organic layer was washed with brine and dried over anhydrous sodium sulfate; the solvent was removed in a vacuum, and the crude product was purified by chromatography (silica gel, 15:1 to 5:1 DCM: MeOH) to give 328 mg (78%) of **CAP-OH** as a white powder. M.P. 73–75 °C.

^1^H-NMR: 6.77–6.84 (m, 3H), 6.07 (s, 1H), 4.34–4.35 (d, J = 5.6 Hz, 1H), 4.31 (s, 1H), 4.14–4.17 (t, 2H), 3.83–3.86 (m, 5H), 3.70–3.72 (m, 2H), 3.64–3.68 (m, 6H), 3.58–3.60 (t, 2H), 2.91–2.93 (t, 2H), 2.17–2.21 (t, 2H), 1.62–1.65 (m, 2H), 1.25–1.28 (m, 10H), 0.84–0.88 (t, 3H).

^13^C-NMR: 173.31, 149.28, 147.01, 132.43, 120.00, 113.49, 111.62, 70.72, 70.51, 70.39, 70.02, 69.53, 68.55, 55.78, 43.00, 40.90, 36.56, 31.78, 29.33, 29.13, 25.83, 22.59, 14.06.

#### Synthesis of CAP-NH_2_

##### 10-azido-*N*-(4-hydroxy-3-methoxybenzyl)decanamide (3)^2^

A solution of vanillylamine hydrochloride (0.600 g, 3.2 mmol) in DMF (2 mL) was placed in a flame-dried round bottom flask under Ar atmosphere. 5 M aqueous solution of NaOH (0.696 mL, 3.48 mmol) was added drop-wise at room temperature. The reaction mixture was stirred for 30 min at 35 °C, cooled to 0 °C, and 10-azidodecanoyl chloride (6 mL, 0.6 M) was added dropwise. The ice bath was removed and the reaction mixture was stirred overnight at room temperature. The reaction was quenched with water (5 mL) and extracted with ethyl acetate (3 × 75 mL). The organic layer was dried over anhydrous sodium sulfate, solvents were removed in a vacuum, and the crude product was purified by chromatography (silica gel; 1:1 to 1:3 hexanes: ethyl acetate) to afford 287 mg (26%) of compound **3** as a colorless oil.

^1^H-NMR: 6.81–6.83 (d, J = 8 Hz, 1H), 6.76 (s, 1H), 6.70–6.72 (dd, J = 8.0, 1.9 Hz, 1H), 6.19 (s, 1H), 6.08 (s, 1H), 4.29–4.31 (d, J = 5.6 Hz, 2H), 3.82 (s, 3H), 3.20–3.24 (t, 2H), 2.15–2.19 (t, 2H), 1.54–1.63 (m, 4H), 1.26–1.31 (m, 10H).

^13^C-NMR: 173.21, 146.91, 145.26, 130.35, 120.74, 114.57, 110.86, 55.97, 51.52, 43.55, 36.80, 29.37, 29.32, 29.30, 29.13, 28.87, 26.74, 25.85.

##### 10-amino-*N*-(4-hydroxy-3-methoxybenzyl)decanamid*e* (CAP-NH_2_)

Triphenyl-phosphine (291 mg, 1.11 mmol) was added to a solution of 10-azido-N-(4-hydroxy-3-methoxybenzyl) decanamide (**3**, 276 mg, 0.792 mmol) in tetrahydrofuran (THF) (2 mL). After 10 min, deionized water (20 µL, 1.11 mmol) was added to the flask, and the reaction mixture was stirred overnight at room temperature. The reaction mixture was concentrated in a vacuum, and the residue was purified by chromatography (silica gel; 15:1 to 1:1 DCM: MeOH) to yield 182 mg (71%) of **CAP-NH**_**2**_ as a light brown oil.

^1^H-NMR: 6.80–6.85 (m, 2H), 6.74–6.76 (d, J = 8.0 Hz, 1H), 5.79 (s, 1H), 4.34–4.36 (d, J = 5.6 Hz, 2H), 3.86 (s, 3H), 3.16 (s, 2H), 2.66–2.70 (t, 2H), 2.17–2.21 (t, 2H), 1.62–1.65 (m, 2H), 1.40–1.44 (m, 2H), 1.25 (s, 10H).

^13^C-NMR: 173.07, 147.35, 145.65, 130.42, 120.91, 115.06, 111.12, 56.06, 43.68, 42.22, 37.02, 33.56, 29.57, 29.48, 29.44, 29.37, 26.97, 26.01.

### Antibodies

Anti-β-actin was from Santa Cruz Biotechnology (sc-47778); anti-Hsp70 from Enzo Life Sciences (catalog no. ADI-SPA-810); anti-Hsc70 from StressMarq Biosciences Inc (catalog no. SMC-151) or anti-Hsp70 (BB70, Dr. David Toft, Mayo Clinic, Rochester, MN); anti-Hsp90β (H90.10, Dr. David Toft); anti-LC3B (Cell Signaling Technology, 3868S);, anti-cleaved LC3A (LC3AII) (Abgent Antibody, Abcepta, San Diego, CA, AP1805a); anti-GAPDH (Santa Cruz Biotechnology, sc-166545); and p62/SQSTM1 from Santa Cruz Biotechnology, Inc (catalog no. sc-28359).

### Chemicals

17-AAG (Selleckchem), Capsaicin (Cayman Chemical) and Ethidium Homodimer-1 (Thermo Fisher).

### Protein purification

Bacterial Hsp90 (HtpG) was expressed in *E. coli* and purified as previously described^88^. Yeast Hsp90 (Hsp82), and cytosolic human Hsp90α and β were expressed in insect sf9 cells and purified as previously described^89^.

### Cell proliferation assays and drug treatments

Cell proliferation was monitored using The CellTiter 96 AQueous One Solution Cell Proliferation Assay (MTS) (Promega #G3580). Cells were grown to 40–60% confluency on 96-well tissue culture plates (Corning #3599, Glendale, AZ) followed by treatment with capsaicin or DMSO control. For protein analysis by Western blot, cancer cell lines were grown for at least 48 h to be at 40–60% confluent in 6-well plates (Corning #3516) before drug treatment was started. Cells were then harvested at specified times, and cell lysates were made. For Western blotting, 15 μg of protein lysate was analyzed using the following specific antibodies.

### Cell lines

HeLa. MCF7, Hs578T, U2OS, LnCaP and PC3 cell lines were purchased from American Type Culture Collection (ATCC, Manassas, VA) within the last 5 years. Large stocks were made and stored in liquid nitrogen. All cell lines are regularly tested at least quarterly for mycoplasma using the Genetica Inc mycoplasma test and MycoAlert PLUS Mycoplasma Detection Kits.

### Western blotting

Cells were lysed with buffer A (10 mM Tris pH 8.0, 137 mM NaCl, 10% glycerol, 1% Nonident P-40) supplemented with a protease inhibitor cocktail (Roche Applied Science, catalog no. 11 836 170 001) on ice for 30 min, shaking every 5 min. The lysate was centrifuged at 16,000×*g* for 10 min. Clarified lysates (15 μg) were run on SDS-PAGE (10% gel) and transferred to PVDF membranes. Proteins were detected by Western blotting using indicated antibodies against specific proteins. All primary antibodies were used at 1/1000 dilution with overnight incubation at 4 °C.

### Spheroid formation assay

The general protocol described by Edner et al. was generally followed^90^. Stable LNCaP and DU145 cell lines containing control or UNC45A shRNA were seeded in a low-adhesion 96-well plate at a density of 5 × 10^3^ cells/well, while stable control and UNC45A sh RNA PC3 cells were seeded at a density of 1 × 10^4^ cells/well. The cells were cultured in serum- and antibiotic-free medium supplemented with B27, N2, insulin, transferrin, selenium, hEGF, and FGF for 8 days. After this time, cells were stained with 0.5 µM EthD-1 and (1:5000 dilution) of Hoechst dyes to distinguish between dead and live cells. Confocal microscopy was used to capture images of the cell spheroids. *t*-test was used for unpaired analysis comparing average expression between conditions. *p* values < 0.05 were considered statistically significant^90^.

### Immunocytochemistry and fluorescence microscopy

The indicated cells were grown in 24-well plates (Corning #3337) on micro-cover glasses (Electron Microscopy Sciences) to about 50% confluency in MEM, 1X (Cellgro #10–010-CV) medium supplemented with 10% fetal bovine serum. Cells were treated with indicated concentrations of 17-AAG, capsaicin, or DMSO control for 24 or 48 h. Cells were fixed with 0.1 M PIPES, pH 6.95; 1 mM EGTA, pH 8.0; 3 mM MgSO_4_; and 3% paraformaldehyde, then permeabilized with 0.1% triton X-100, blocked with 2% fetal bovine serum, 5% goat serum and 5% glycerol, and stored at 4 °C. The primary antibodies against LC3B, as well as mouse and rabbit secondary antibodies, were prepared in the blocking buffer.

### Microscopy

In each well of a 6-well tissue culture plate, 100,000 MCF7 cells were grown to 60% confluence. Cells were treated with 200 μM capsaicin or DMSO control (1.5% total DMSO concentration) for 15 h. Cells were fixed and imaged using a light microscope (Carl Zeiss). Mr. Brendan Marshall from the Electron Microscopy & Histology Core Laboratory at Augusta University performed the electron microscopic study of MCF7 cells.

### Immunoprecipitation from cell lysates

Monoclonal Hsp70 antibody (BB70) was incubated with 30 μL slurry of protein A/protein G-agarose beads (Pierce Protein A Agarose, # 20334 and Pierce Protein G Agarose, # 20399) for 2 h at room temperature. Following incubation, cell extracts (200 μg of protein) from MCF7 cells were added to each sample and incubated overnight at 4 °C with gentle rotation. The samples were then washed three times with 1X RIPA buffer along with protease inhibitors (Roche Applied Science, #11836170001), 10 mm NaF, 2 mm sodium pyrophosphate, 2 mm β-glycerophosphate, and 1 mm sodium orthovanadate, and 1 mM phenylmethylsulfonyl fluoride (PMSF) and eluted with 2× sample buffer at 95 °C for 5 min. Protein complexes were eluted using SDS-PAGE sample buffer and analyzed by Western blot.

### CETSA

To analyze the engagement of capsaicin and Hsp90 protein, a cellular thermal shift assay was performed. 1 × 10^7^ Hs578T cells were seeded in 10 cm culture dishes. After 24 h, cells were treated with DMSO (1%) or capsaicin (200 μM) for 1 h. Following treatment, cells were trypsinized and collected with PBS containing protease inhibitors and transferred to PCR tubes. All samples were heated at 52–60 °C for 2 min using a Bio-Rad C1000 thermal cycler and lysed with 3–4 freeze–thaw cycles using liquid nitrogen. For each freeze–thaw cycle, the cells were exposed to liquid nitrogen for 1 min and placed in a water bath at 30 °C for 1 min. Precipitated proteins and cell debris were then pelleted by centrifugation at 16,400 rpm for 15 min at 4 °C and the cleared cell lysates were analyzed by Western blotting.

### Capsaicin derivative immobilization on Sepharose resin and pull-down experiments

Capsaicin derivatives solubilized in 5% DMSO were covalently linked to Sepharose 4B as described previously by Cornillot et al.^47^. The Cap-NH2 immobilization was implemented at pH 10, and Cap-OH immobilization was done at pH 6. Sepharose resin subjected to Divinyl sulfone activation and blocking with ethanolamine was used as a control. To test the binding of Hsp90 and its fragments to capsaicin derivative resins, 15 µL of resin and 10 µg of protein were used in a final reaction volume of 200 µL of incubation buffer [10 mM Tris, 100 mM KCl, 0.02% NP40, 2.5% glycerol, and 2 mM DTT (pH 7.5)]. Samples were incubated for 30 min at 30 °C and washed four times with 1 ml of buffer. Bound proteins were extracted with sample buffer, resolved by SDS–PAGE, and stained with Coomassie Blue.

### Progesterone receptor (PR) reconstitution assay

Purified PR was adsorbed onto PR22 antibody-protein A-Sepharose resin beads and was assembled into complexes, as described previously^91^. Rabbit reticulocyte lysate (RRL) was used to refold the PR. Briefly, approximately 0.05 μM PR was incubated with 1.4 μM Hsp70, 0.8 μM Hsp90β, 0.2 μM Hsp40 (Ydj), 0.08 μM HOP, and 2.6 μM p23 in reaction buffer (20 mM Tris/HCl, pH 7.5, 5 mM MgCl_2_, 2 mM DTT, 0.01% NP-40, 50 mM KCl, and 5 mM ATP). After incubation for 30 min at 30 °C, 0.1 μM [^3^H]-progesterone (American Radiolabeled Chemicals, Inc, #ART 0063, St. Louis, MO) was added. Samples were incubated on ice for 3 h at 4 °C. Complexes were then washed three times with 1 ml of reaction buffer and assessed for bound progesterone by liquid scintillation counting using a PerkinElmer Microbeta plate reader. PR activity is expressed in radioactive count per minute (CPM).

### Molecular docking

Discovery Studio 2.5 software was used to carry out docking simulations using the Hsp90 crystal structure co-crystalized with the selective inhibitor PU3 (PDB accession number: 1UYM)^68^. The protein and ligand were optimized with force field: CHARMm, Partial charge: MMFF94, and the radius of receptor active site employed: 4.030776, 10.9669, 24.5626, 9.0. Our results showed that the superimposition of the docked poses with the bioactive ligand conformations yields a low root mean square deviation (RMSD) of 0.095 Å, indicating that molecular docking can accurately reproduce the ligand conformation in the Hsp90 crystal structures.

### Supplementary Information

Supplementary Information.

## Data Availability

Raw data generated during this study are included in this published article [and its supplementary information files]. All the raw data generated by mass spectrometry analysis and large files of images used to generate Figs. 4B–D, and 5C are available from the corresponding author on reasonable request.
