# Supplementary material for: Capsaicin binds the N-terminus of Hsp90, induces lysosomal degradation of Hsp70, and enhances the anti-tumor effects of 17-AAG (Tanespimycin)
Source: Sci Rep. 2023 Aug 23;13:13790. doi: 10.1038/s41598-023-40933-9 (PMC10447550; doi:10.1038/s41598-023-40933-9)
Supplement: Supplementary file 1 — Supplementary Information. [file 41598_2023_40933_MOESM1_ESM.pdf]

Title: Capsaicin binds the N-terminus of Hsp90, induces lysosomal degradation of Hsp70, and enhances the anti-tumor effects of 17-AAG (Tanespimycin).

Chaitanya A. Patwardhan[1], Vamsi Krishna Kommalapati[1], Taoufik Llbiyi[1], Digvijay Singh[1], Eyad Alfa[1], Anatolij Horuzsko[1], Hasan Korkaya[1], Siva Panda[2], Christopher A. Reilly[3], Vladimir Popik[4] and Ahmed Chadli[1]

Data availability: Raw data generated during this study are included in this published article [and its supplementary information files]. All the raw data generated by mass spectrometry analysis and large files of images used to generate Figures 4B, C, D, and Figure 5C are available from the corresponding author on reasonable request.

Raw data

Figure 1

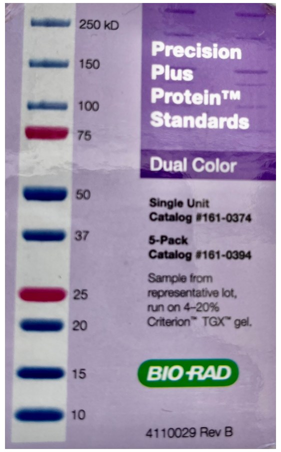

Protein standards used throughout this study

Figure 1A

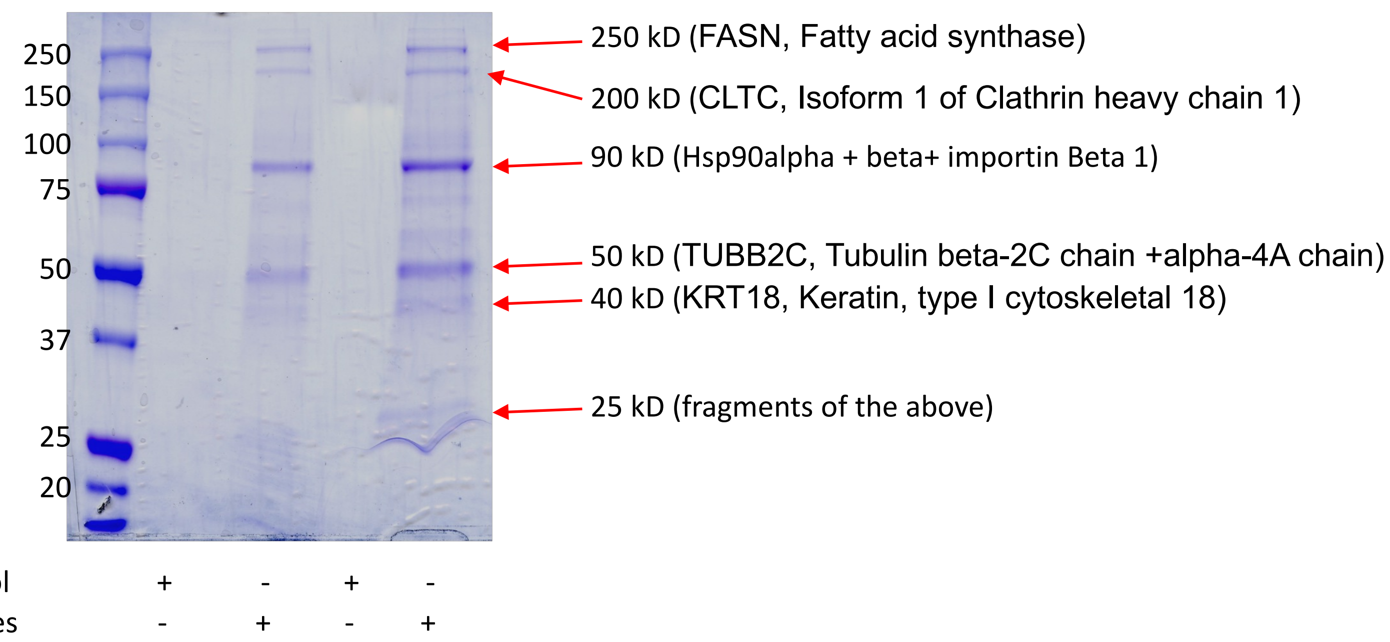

Figure 1C

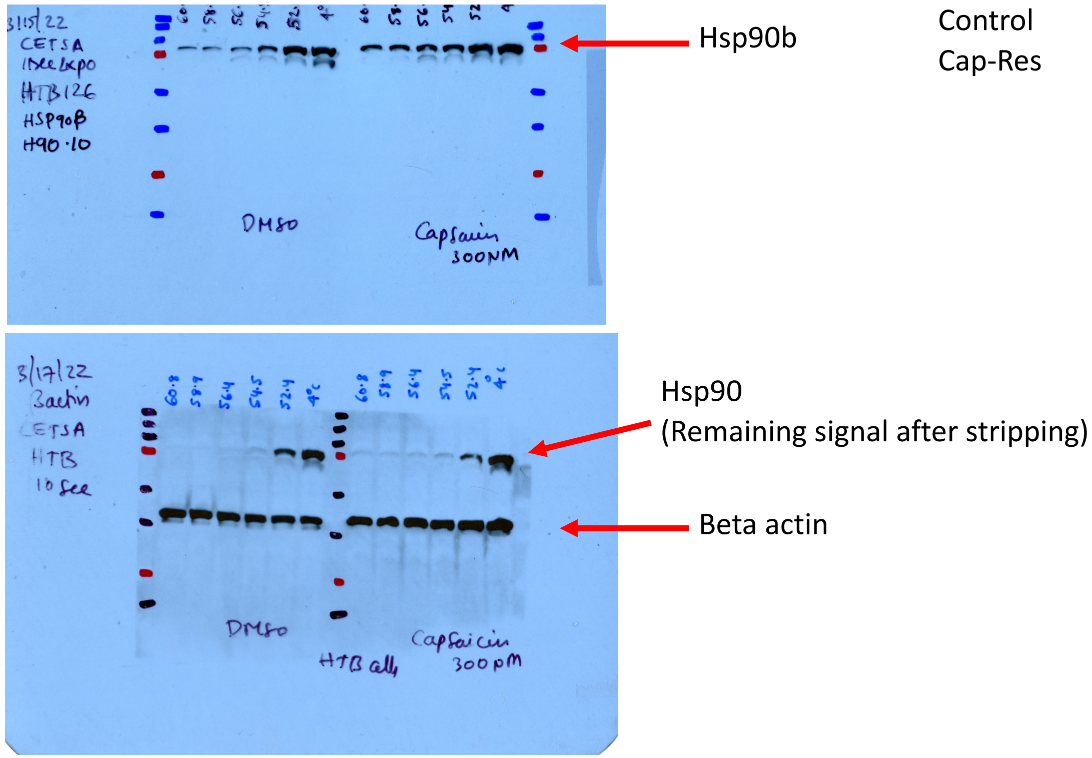

Figure 1D

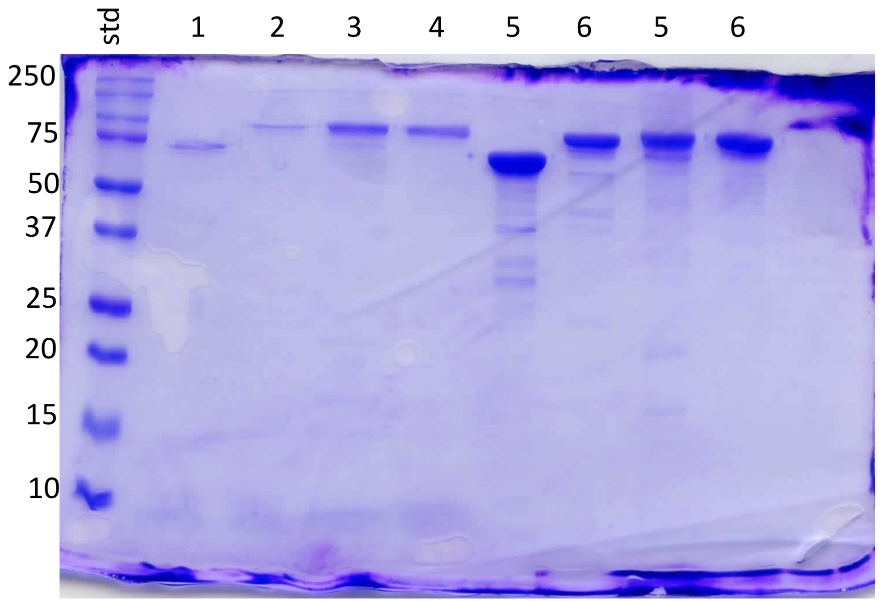

1: HTPG

2: YHsp82

3: Hsp90a

4: Hsp90b

5: HTPG

6: YHsp82

7: Hsp90a

8: Hsp90b

Quantification of Figure 1D.

	Bound	Loading	IP/Loading
HtpG	13555.484	143905.094	0.09419739
YHsp82	12988.2	94053.149	0.13809426
Hsp90α	69234.818	119126.161	0.58118903
Hsp90β	41883.848	125269.454	0.33435005

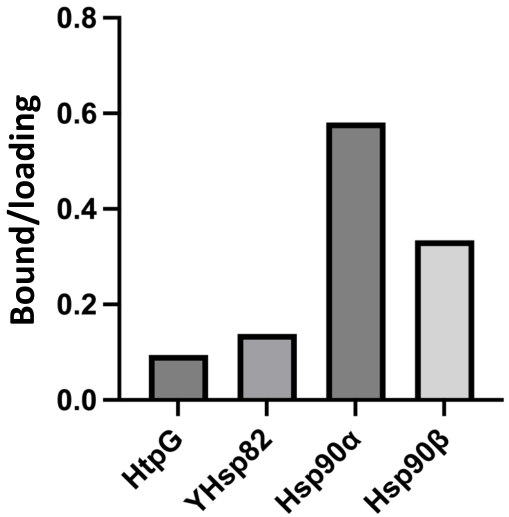

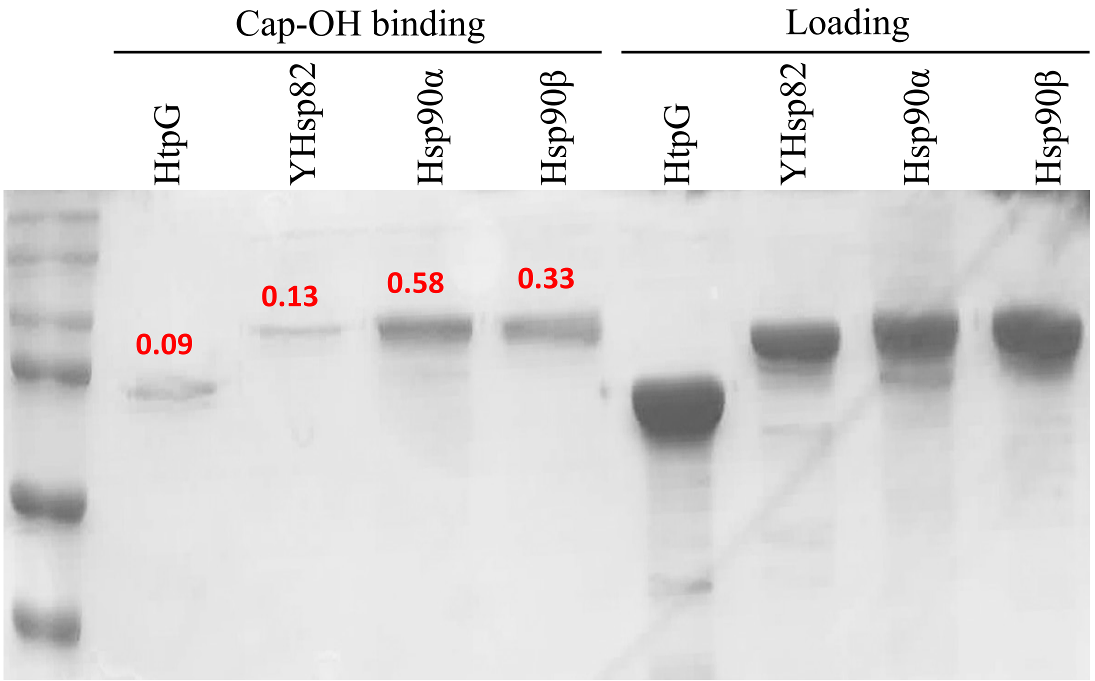

Hsp90 orthologs binding to capsaicin resin. Relative density was calculated by dividing bound protein signals with the corresponding loading control signal using NIH Image J software.

Raw data
Figure 2

Figure 2A

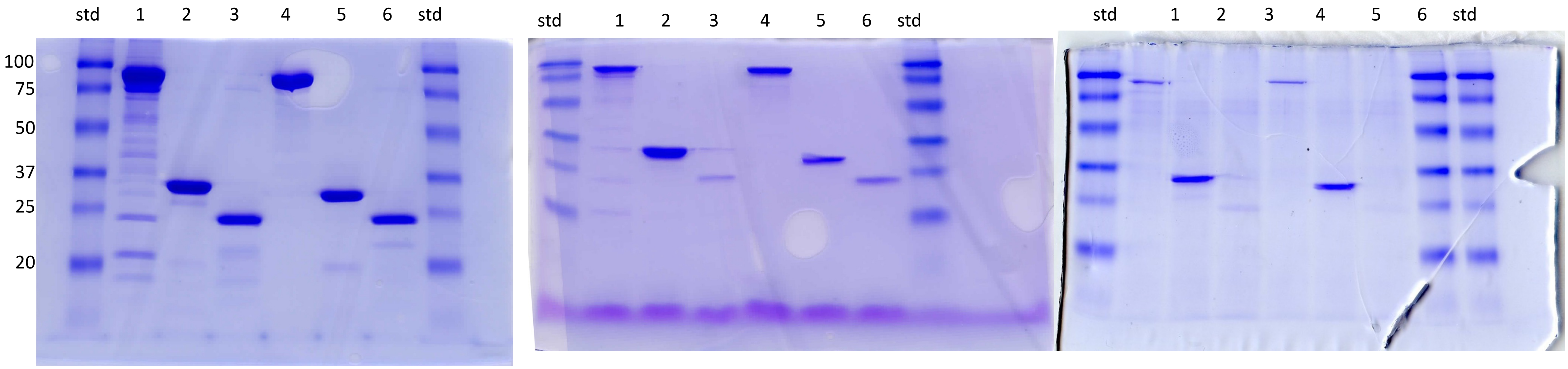

Loading

Cap-OH

Cap-NH2

1: Hsp9a FL

2: Hsp90a 1-212

3: Hsp90a 616-Ct

4: Hsp9b FL

5: Hsp90b 1-212

6: Hsp90b 616-Ct

Quantification of Figure 2A

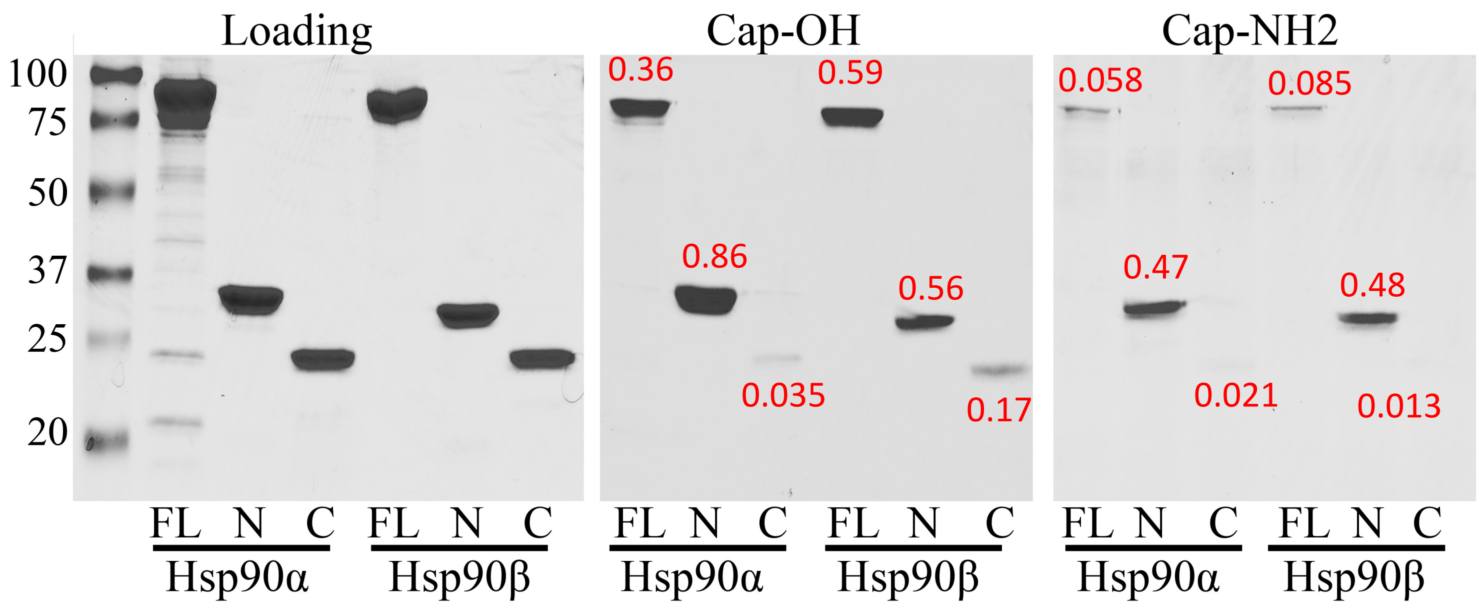

	Hsp90α Cap-OH	Hsp90β Cap-OH	Hsp90α Cap-NH2	Hsp90β Cap-NH2
FL	0.36507646	0.59291344	0.0584625	0.0859557
N	0.86888642	0.56355437	0.47532237	0.48276535
C	0.03545048	0.17319652	0.02161596	0.01369814

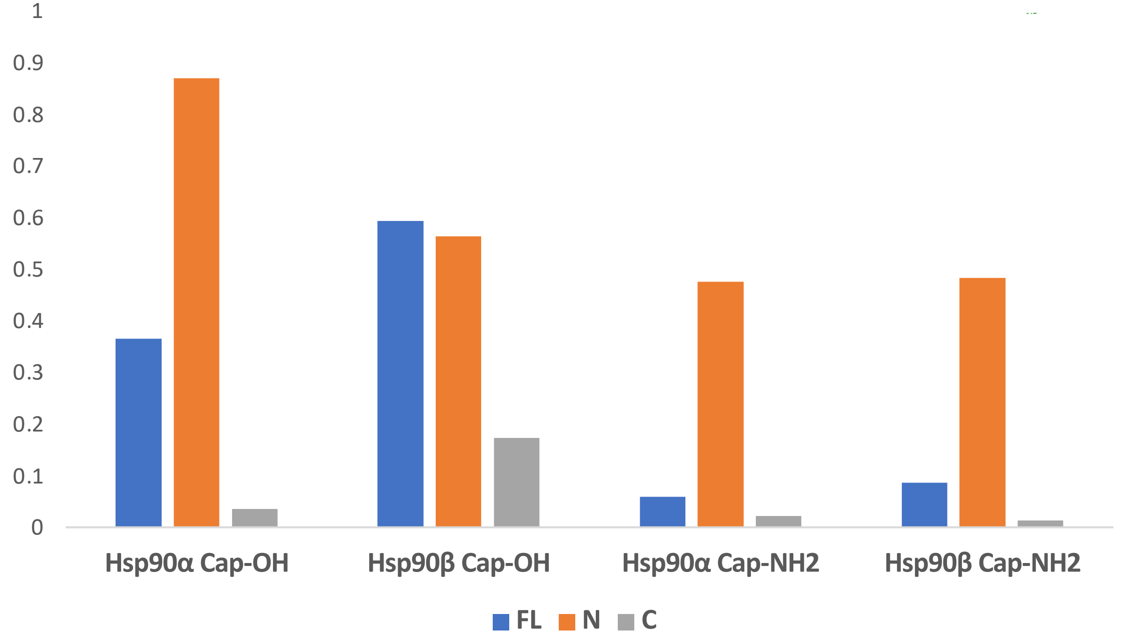

Hsp90 domains binding to capsaicin resins. Relative density was calculated by dividing bound protein signals with the corresponding loading control signal using NIH Image J software.

Raw data
Figure 3

Figure 3A

HeLa

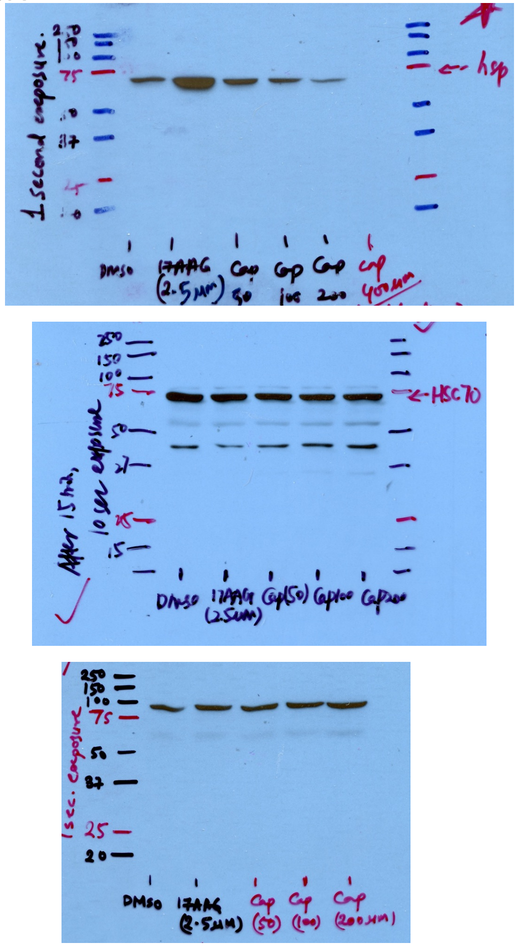

MCF7

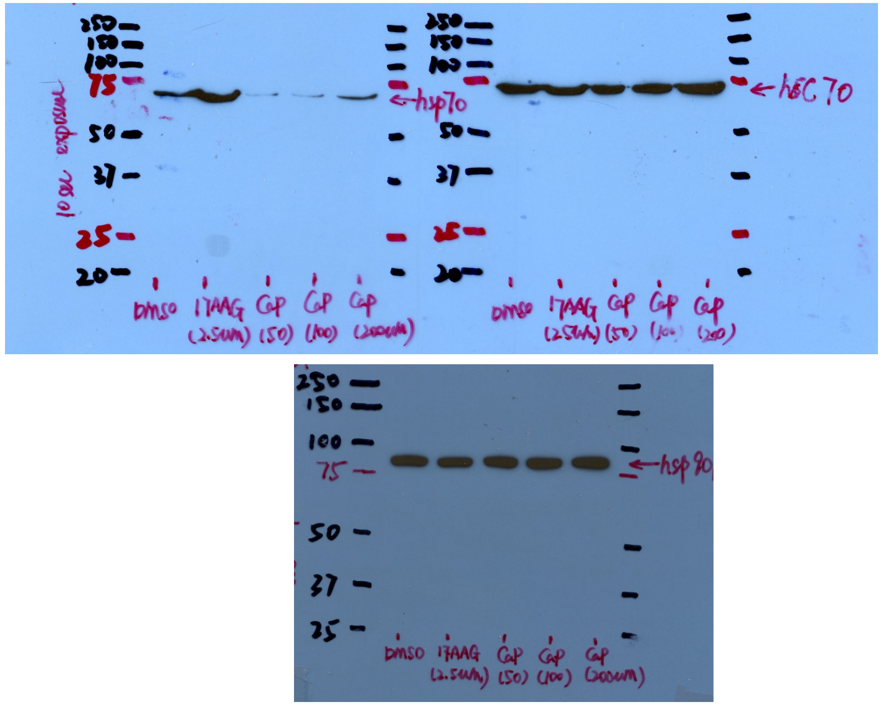

U2OS

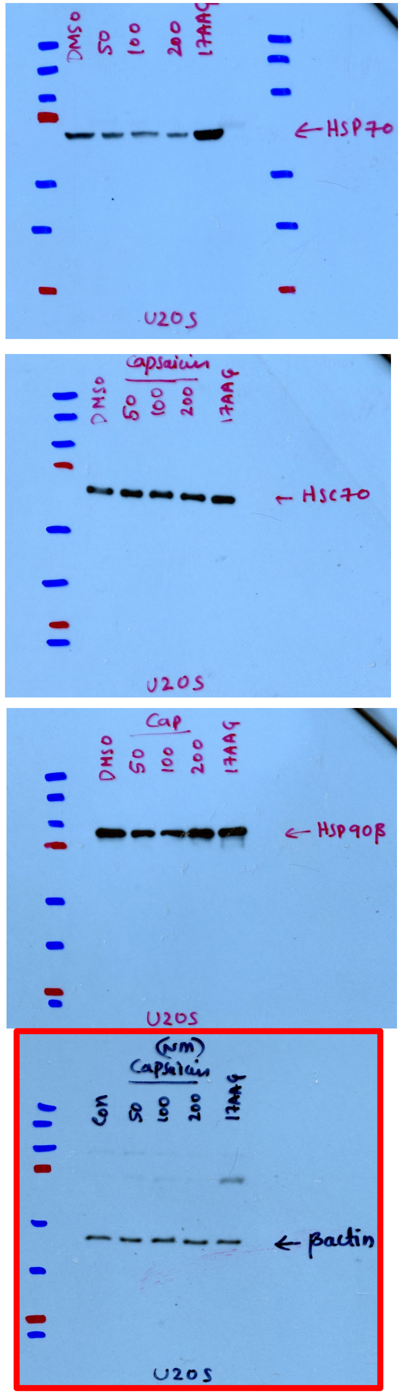

LnCaP

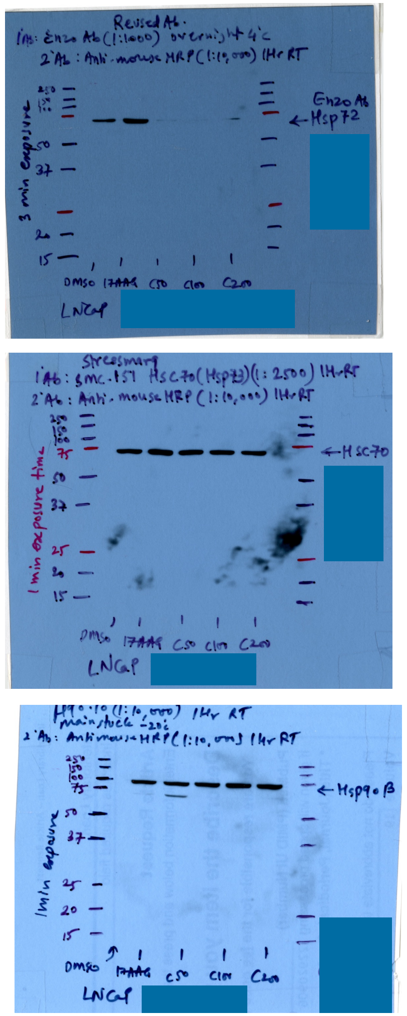

Raw data
Figure 3

Figure 3C

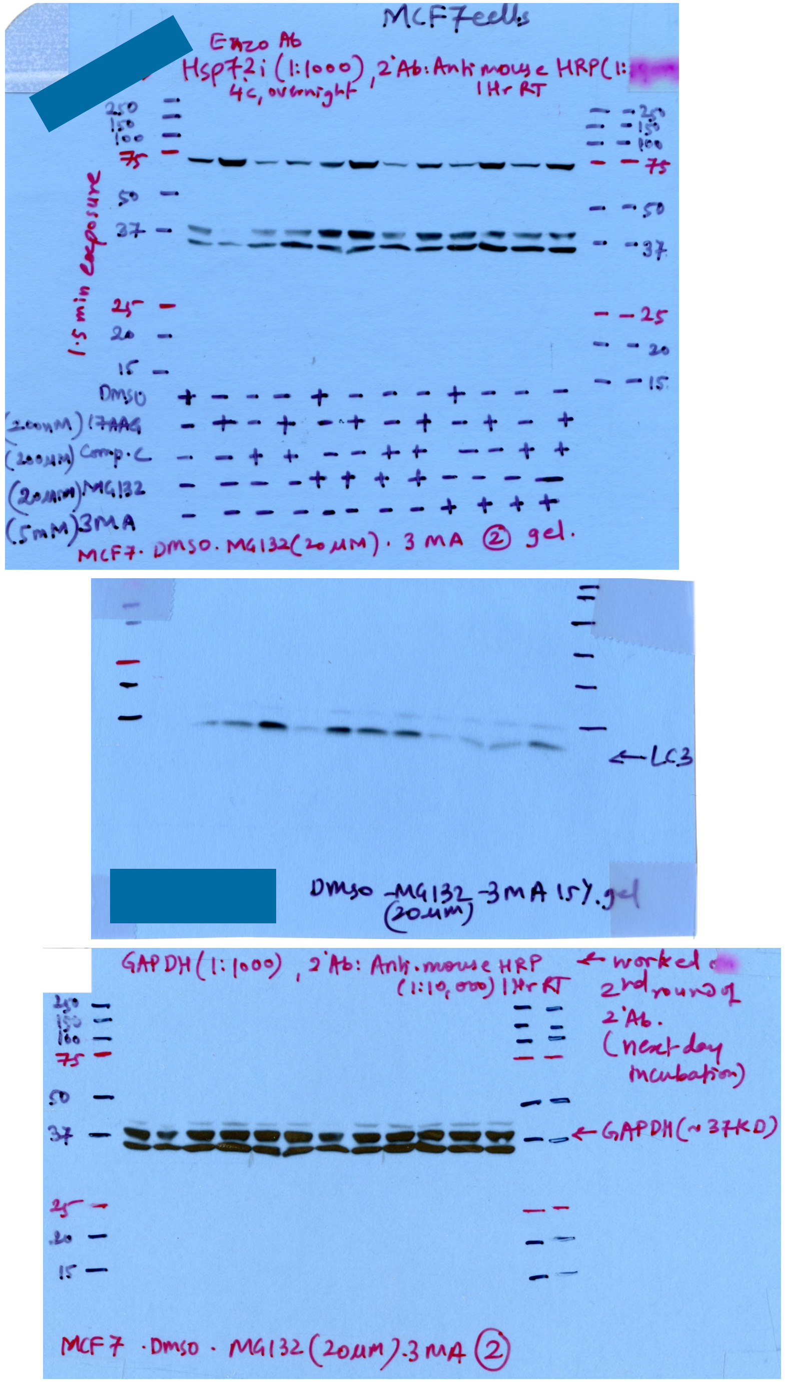

Figure 3B

Not shown in Figure 3C

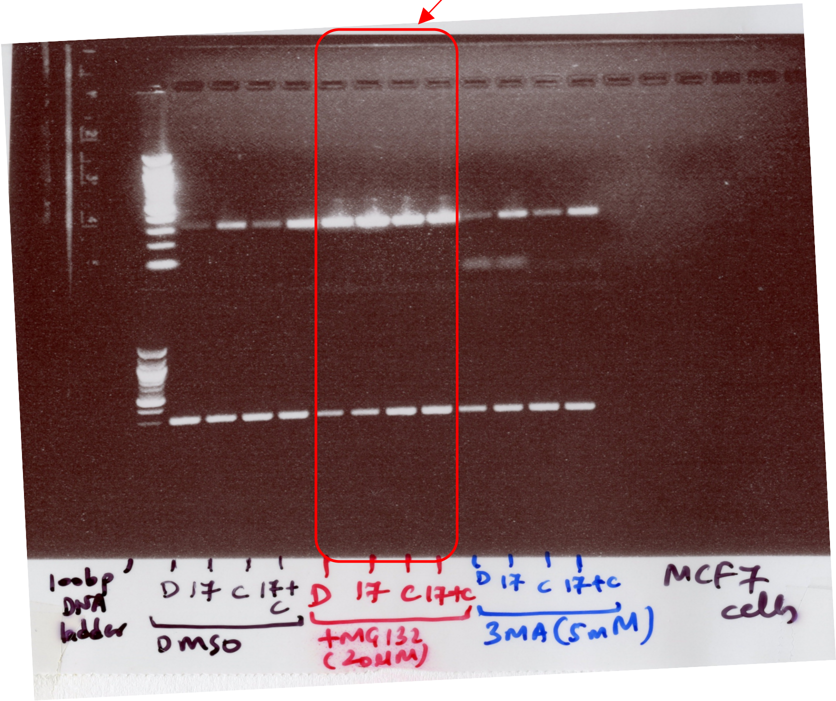

Quantification of Figure 3C

		Hsp70	actin	Hsp70/actin
1	DMSO	48833.099	92895.12	0.52567992
2	17AAG	109944.392	55914.492	1.96629511
3	Capsaicin	14065.815	103484.869	0.13592147
4	CAP+17AAG	26921.321	109426.513	0.24602192
5	DMSO	23788.451	90278.442	0.2635009
6	17AAG	63915.179	97589.362	0.65494002
7	Capsaicin	7430.995	69805.856	0.10645232
8	CAP+17AAG	26359.421	90882.999	0.29003687
9	DMSO	17997.17	79548.434	0.22624166
10	17AAG	96720.312	86367.253	1.11987251
11	Capsaicin	35148.442	108964.927	0.32256656
12	CAP+17AAG	98934.555	91216.626	1.08461099

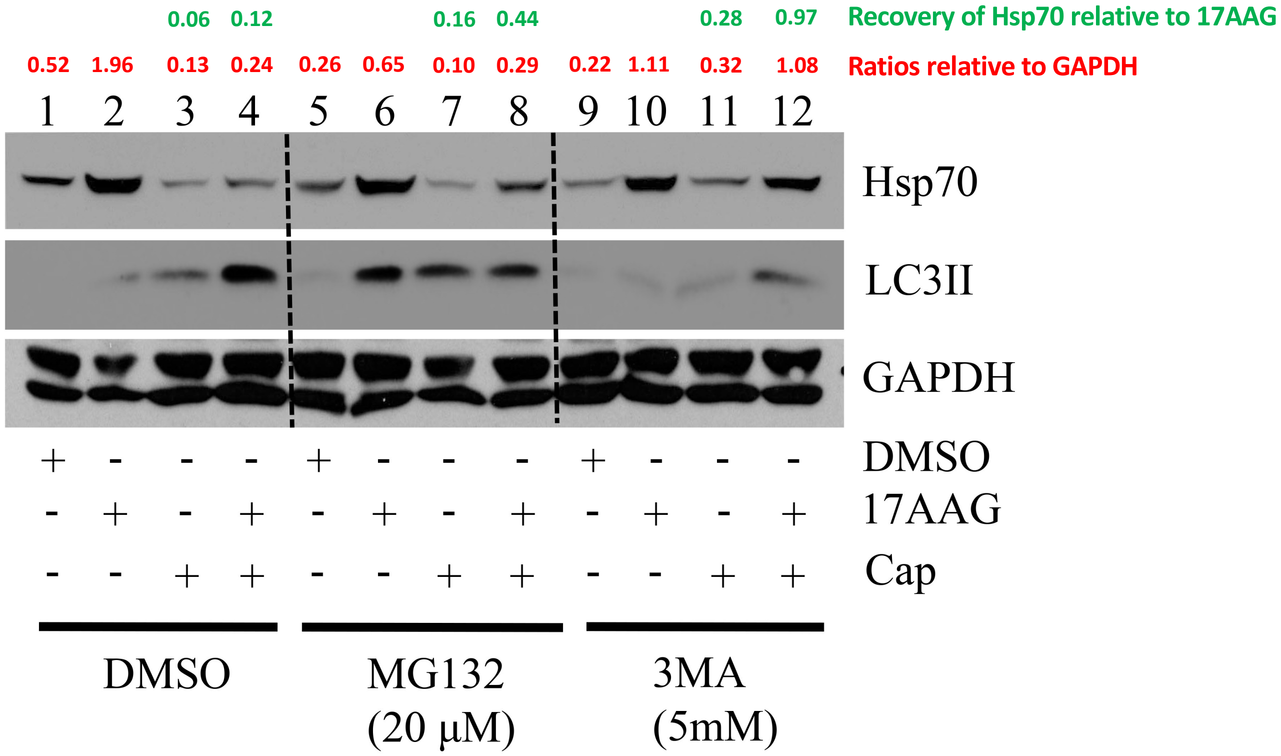

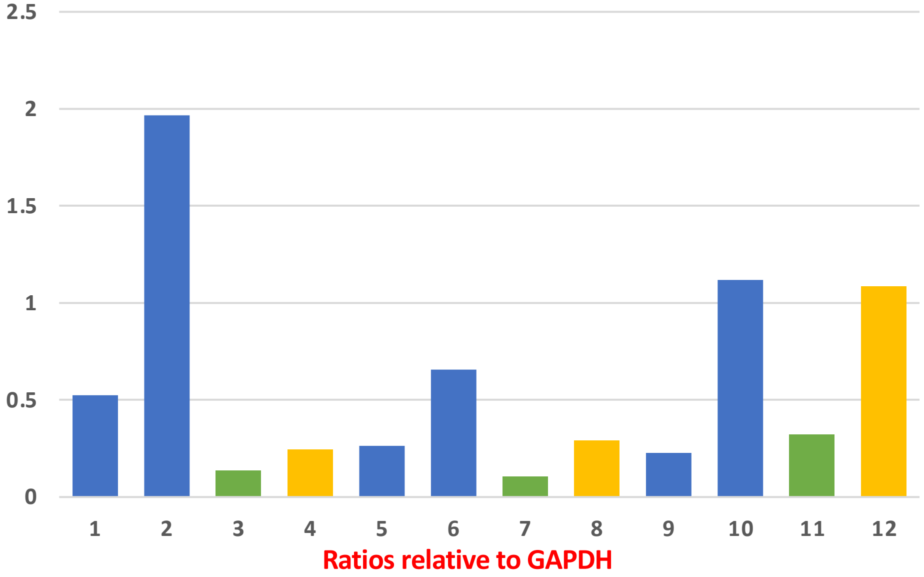

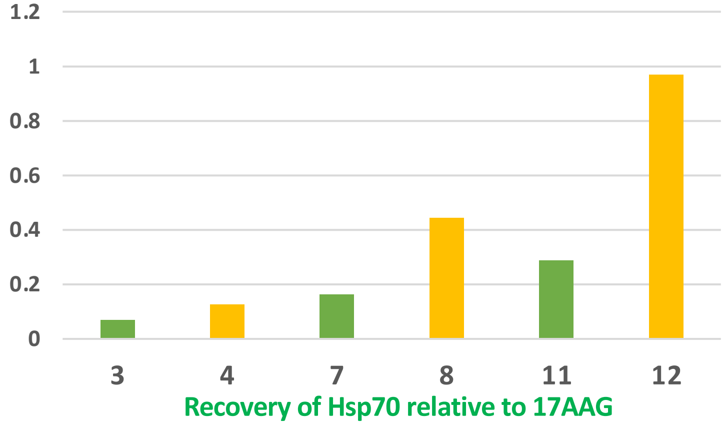

Quantification of Hsp70 recovery upon treatment with MG132 and 3MA. Hsp70 relative density was calculated by dividing the Hsp70 signal by the corresponding GAPDH signal. The recovery rate was calculated by dividing the relative Hsp70 signal by the Hsp70 rate obtained for 17-AAG. NIH Image J software was used to quantify the bands.

Raw data

Figure 4

Figure 4A

HeLa data are not shown

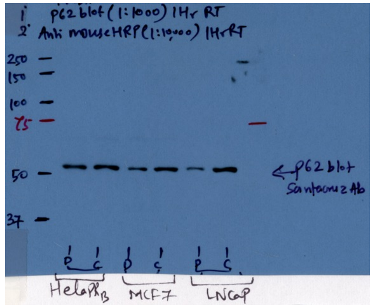

p62

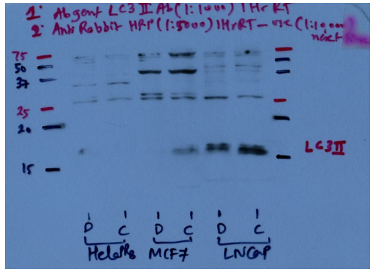

Low exposure

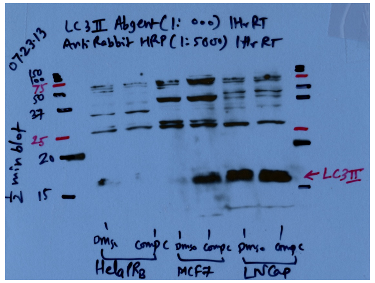

High exposure

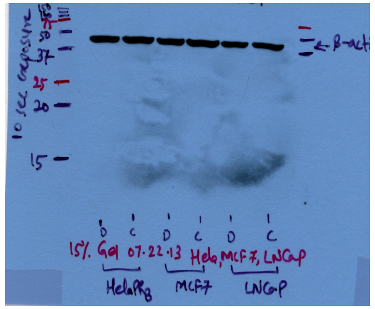

Beta actin

Figure 4C

DMSO

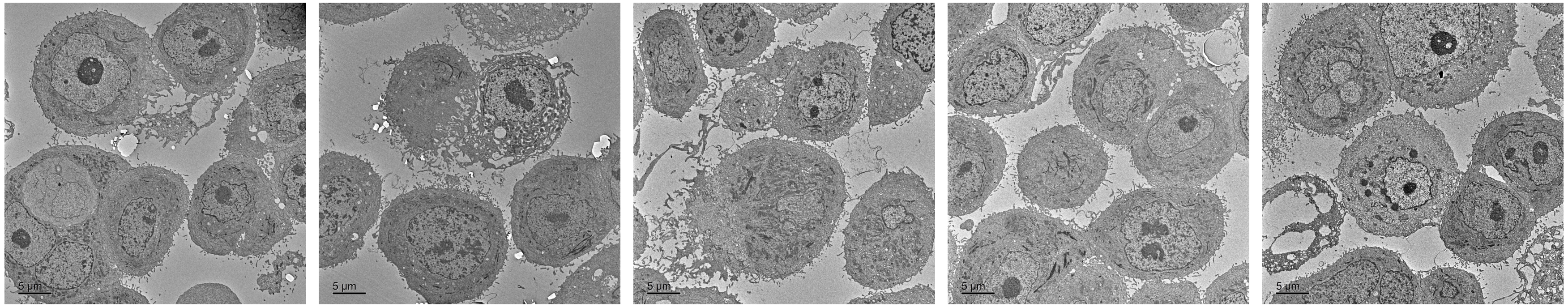

Capsaicin

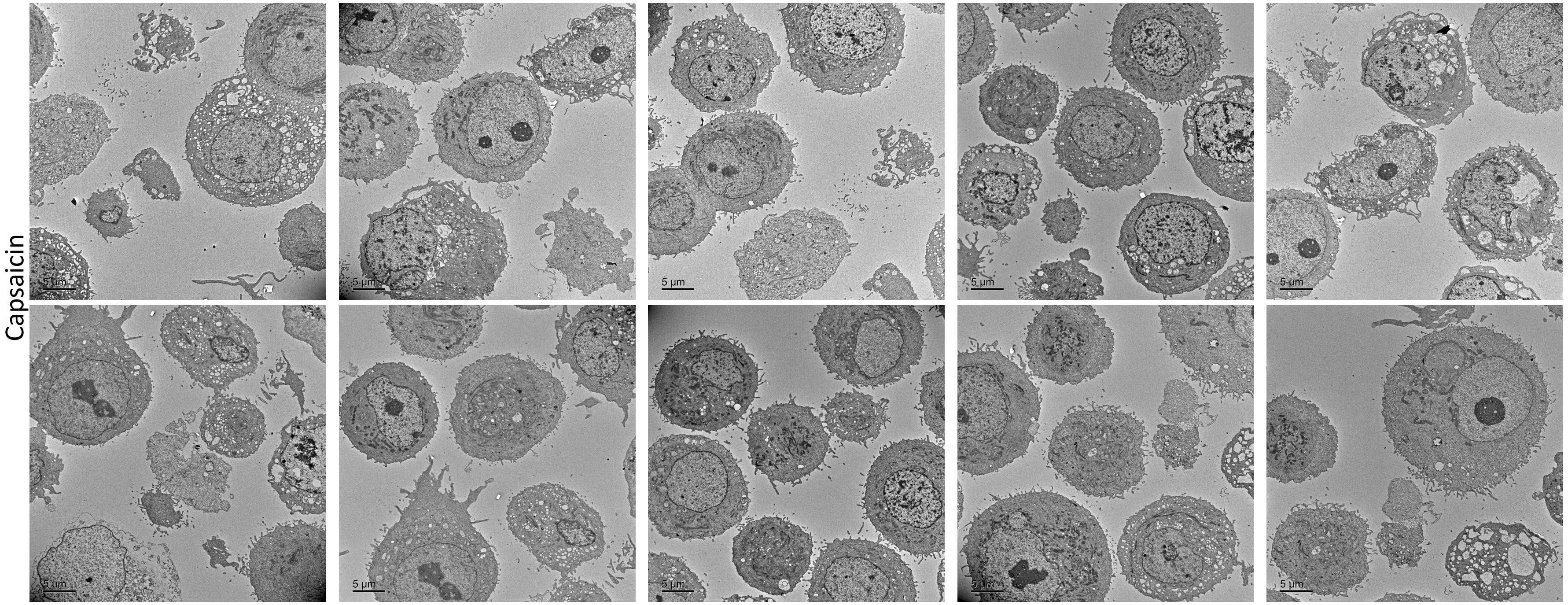

Raw data
Figure 5

Figure 5A

Purified Hsp70 used as a reference

MCF7

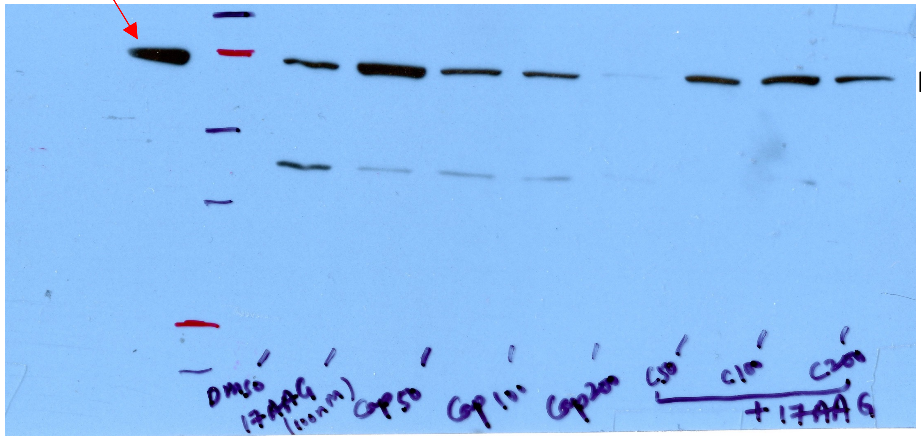

Hsp70

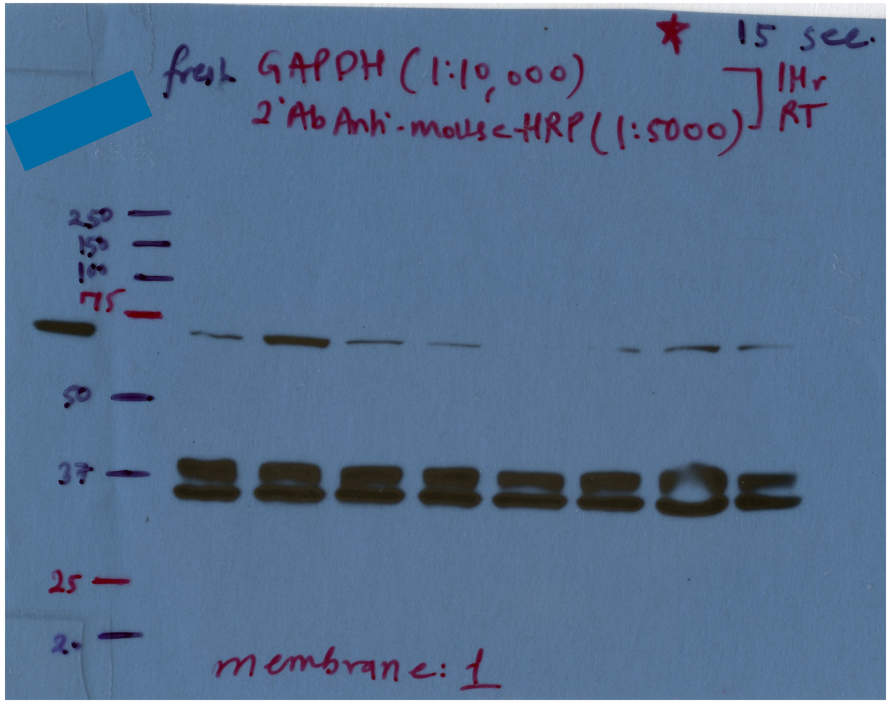

Hsp70
(Remaining signal after stripping)

GAPDH

LnCaP

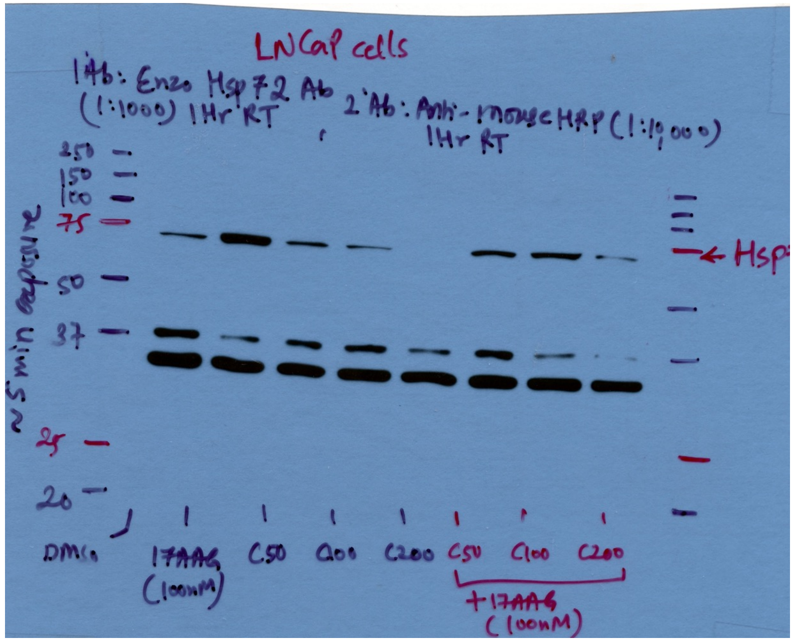

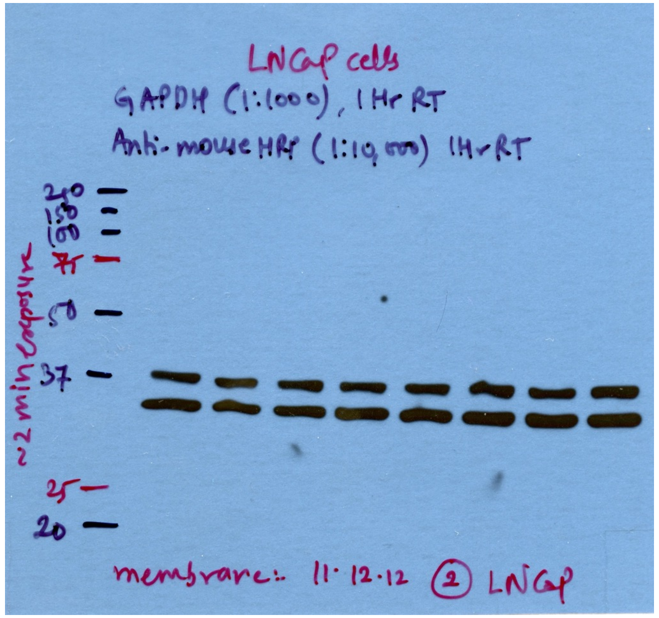

	MCF7	Hsp70	GAPDH	Hsp70/GAPDH	Hsp70 relative to 17AAG
1	DMSO	73221.765	124672.927	0.58731087	0.33044473
2	17AAG	176632.141	99380.35	1.77733466	1
3	Cap 50	74932.856	106830.563	0.70141778	0.39464587
4	Cap 100	56970.685	117295.735	0.48570125	0.27327507
5	Cap 200	4715.2	132914.735	0.03547537	0.01995987
6	17AAG+ Cap 50	76729.35	138392.392	0.5544333	0.31194648
7	17AAG+ Cap 100	102752.563	152137.584	0.67539237	0.38000292
8	17AAG+ Cap 200	60290.17	95625.806	0.63048012	0.35473349
	LNCAP	Hsp70	GAPDH	Hsp70/GAPDH	Hsp70 relative to 17AAG
1	DMSO	48356.978	115861.999	0.41736703	0.34520279
2	17AAG	165510.584	136893.22	1.2090488	1
3	Cap 50	51831.421	121714.664	0.42584368	0.35221381
4	Cap 100	26324.321	137244.463	0.19180607	0.15864212
5	Cap 200	4953.362	148708.17	0.03330928	0.02754999
6	17AAG+ Cap 50	76182.978	143177.856	0.53208632	0.44008671
7	17AAG+ Cap 100	91746.785	152268.413	0.60253327	0.49835314
8	17AAG+ Cap 200	22050.693	152445.362	0.14464653	0.11963664

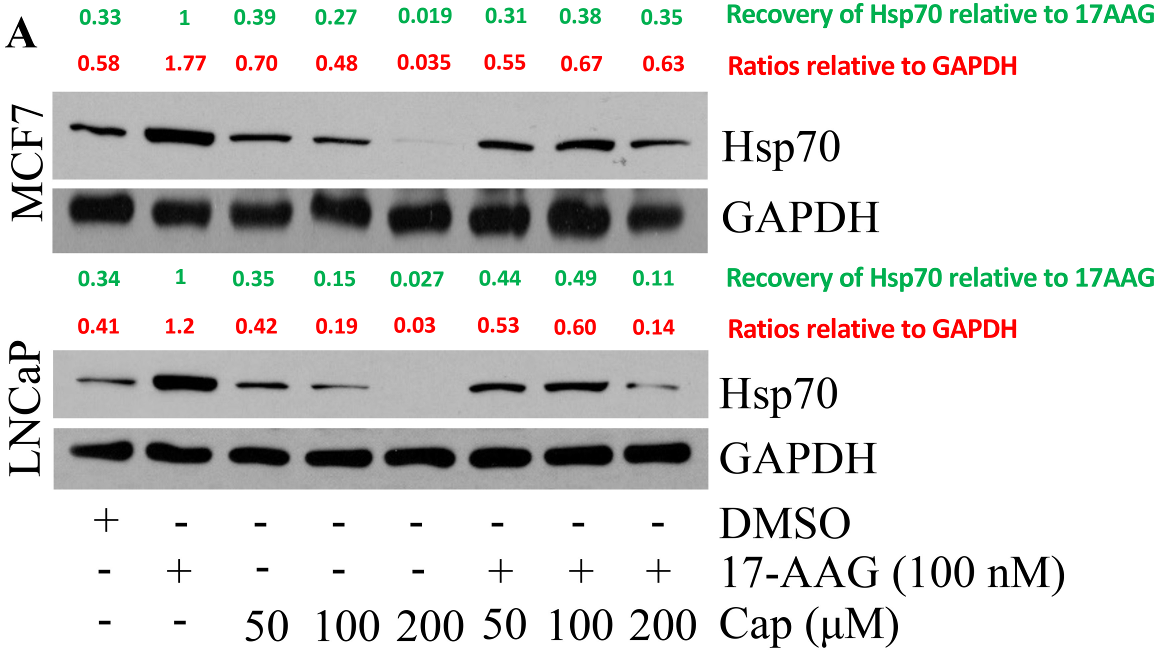

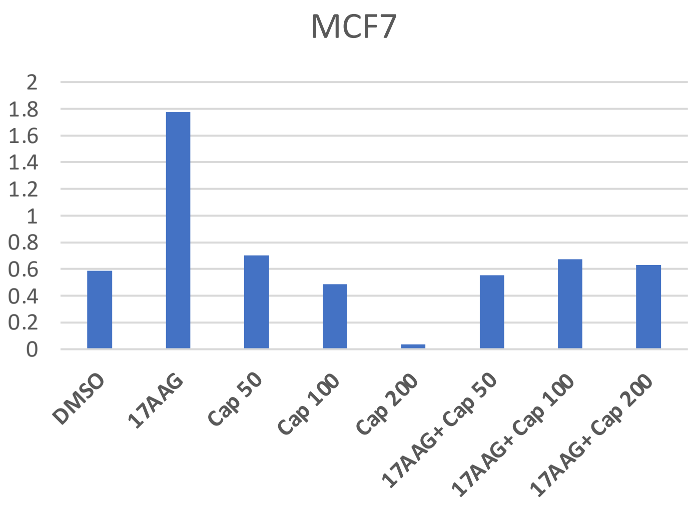

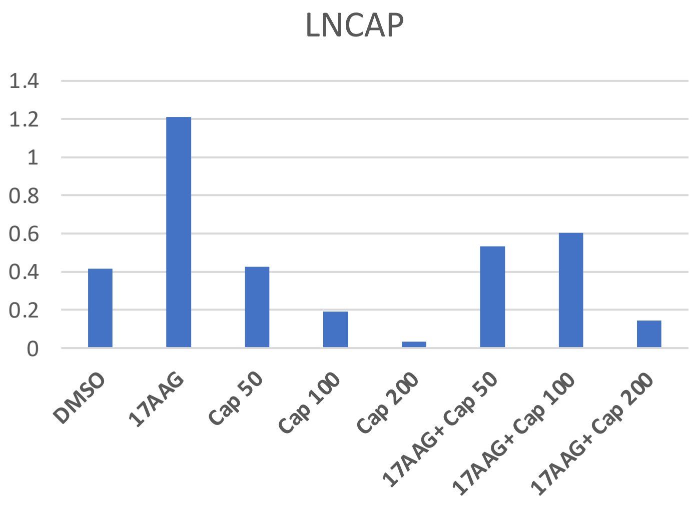

Quantification of Hsp70 overexpression upon treatment with capsaicin alone or in combination with 17-AGG. Hsp70 relative density was calculated by dividing the Hsp70 signal by the corresponding GAPDH signal. NIH Image J software was used to quantify the bands.
